# Novel Coumarin-Containing Aminophosphonatesas Antitumor Agent: Synthesis, Cytotoxicity, DNA-Binding and Apoptosis Evaluation

**DOI:** 10.3390/molecules200814791

**Published:** 2015-08-13

**Authors:** Ya-Jun Li, Cai-Yi Wang, Man-Yi Ye, Gui-Yang Yao, Heng-Shan Wang

**Affiliations:** 1 State Key Laboratory Cultivation Base for the Chemistry and Molecular Engineering of Medicinal Resources, School of Chemistry & Chemical Engineering of Guangxi Normal University, Guilin 541004, China; E-Mails: liyajungxnu@163.com (Y.-J.L.); baobei_mayu@163.com (M.-Y.Y.); 2 College of Medicine and Pharmacy, Hunan Polytechnic of Environment and Biology, Hengyang 421000, China; 3 College of Chemical and Material Science, Hebei Normal University, Shijiazhuang 050024, China; E-Mail: wangcaiyigxnu@163.com

**Keywords:** 7-hydroxy-4-methylcoumarin, α-aminophosphonates, synthesis, cytotoxicity, cell cycle, DNA binding

## Abstract

A series of novel coumarin-containing α-aminophosphonates were synthesized and evaluated for their antitumor activities against Human colorectal (HCT-116), human nasopharyngeal carcinoma (human KB) and human lung adenocarcinoma (MGC-803) cell lines *in vitro*. Compared with 7-hydroxy-4-methylcoumarin (**4-MU**), most of the derivatives showed an improved antitumor activity. Compound **8j** (diethyl 1-(3-(4-methyl-2-oxo-2H-chromen-7-yloxy) propanamido)-1-phenylethyl-Phosphonate), with IC_50_ value of 8.68 μM against HCT-116 cell lines, was about 12 fold than that of unsubstituted parent compound. The mechanism investigation proved that **8c**, **8d**, **8f** and **8j** were achieved through the induction of cell apoptosis by G1 cell-cycle arrest. In addition, the further mechanisms of compound **8j**-induced apoptosis in HCT-116 cells demonstrated that compound **8j** induced the activations of caspase-9 and caspase-3 for causing cell apoptosis, and altered anti- and pro-apoptotic proteins. DNA-binding experiments suggested that some derivatives bind to DNA through intercalation. The results seem to imply the presence of an important synergistic effect between coumarin and aminophosphonate, which could contribute to the strong chelating properties of aminophosphonate moiety.

## 1. Introduction

Chemical modification of bioactive components of medicinal herbs is one of the most common approaches in drug discovery for new drugs and improved therapeutic properties. Coumarins are an important class of compounds obtained from nature and synthetic origin that possess diverse pharmacological and biological activities such as antitumor [1], anti-inflammatory [2], anticoagulant [3], anti-HIV [4], herbicidal [5] and fungicidal [6] activities. The pharmacological and biochemical properties of coumarins depend upon the pattern of substitution of the naturally occurring scaffold. The naturally occurring 7-hydroxy coumarin derivatives have been investigated as potential lead structures for cancer drug development [7]. Some 4-hydroxycoumarins, bearing an aryl group in the third position, inhibit cancer cell proliferation [8].

Novobiocin, a member of amino-4-hydroxycoumarin family, has been shown to possess anticancer activity, owing to its amide side chain, the coumarin ring and the sugar moiety [9]. However, little attention has been paid to the cytotoxicity and DNA binding affinity studies of **4-MU**, which belongs to the same class of coplanar coumarin as novobiocin [10]. In general, most DNA binding compounds containing a linear or angular planar chromophore with apolyaromatic ring can influence the structures and physiological functions of DNA [11]. To enhance antitumor activity, generally the DNA binding compounds should carry one or two flexible basic side chains on chromophore [12]. The flexible basic side chains are highly influential in directing the thermodynamic-binding mechanism, geometry of the ligand-DNA complex and sequence selectivities [13].

Recently, Łukasz Berlicki and his co-workers [14] suggested that the N-C-P molecular fragment and its chemistry could offer many possibilities for structural modifications, which have resulted in broad biological relevance. As an important class of compounds containing N-C-P molecular fragment, α-aminophosphonates (APAs) and their derivatives are an important class of compounds that exhibit intriguing biological activities [15], especially antitumor [16] and inhibitors of enzyme related to tumor genesis and invasions [17]. Because alkaline phosphatase is known to be over expressed in the extracellular space of specific tumor cells such as ovarian and hepatic carcinoma cells, introducing a phosphate group for targeted delivery appears to be a reasonable strategy to increase solubility and enhance transport through cellular membrane [18]. Some phosphate groups also exhibit high affinity to calcium ions and have been used to design targeted drugs for bone cancer [19]. Since phosphonate esters can be hydrolyzed under biological conditions, aminophosphonate esters are a good choice in designing targeted anticancer drug [20].

Recently, it was found that APAs group could enhance the antitumor activities of coumarin in our laboratory [21]. As part of our series of research work, coumarin-bearing α-aminophosphonates were designed in our recent study to establish more efficient antitumor lead compounds. This work aims to synthesize a group of new coumarin derivatives with improved DNA binding affinity and antitumor activity by incorporating various α-aminophosphonates into the chromophore. The DNA binding capacity of the new coumarin derivatives was evaluated and their cytotoxicity was assessed on three types of tumor cell lines. Flow-cytometric analysis was also employed in the study to reveal the mechanism of how some compounds showing higher activity killed HCT-116 cells.

## 2. Results and Discussion

### 2.1. Synthesis of Coumarin Derivatives

**Scheme 1:**
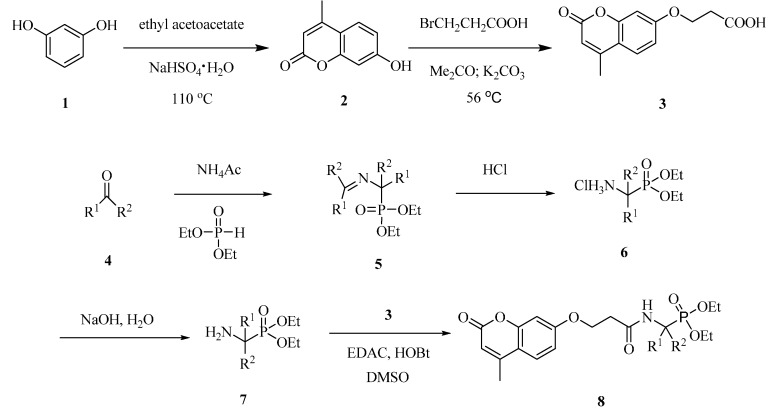
General synthetic route for compound **8a**–**8j**.

The synthetic steps of the target compounds are shown in Scheme 1. The synthesis of 7-hydroxy-4-methylcoumarin **2** was carried out according to our previous work [21], which included the condensation of phenols with ethyl acetoacetate in the presence of the catalysis sodium bisulfate. Compound **3** was then obtained in good yields by the coupling reaction of **2** with 3-bromopropionic acid in the presence of potassium carbonate.

*O*,*O*′-dialky ((*N*-(phenylmethylene)-α-amino)-α-(substituted phenyl)methyl) phosphonates **5**, obtained by reacting substituted benzaldehyde **4** with ammonium acetate and *O*,*O*′-dialkylphosphite, were converted to *O*,*O*′-dialkylα-amino-(α-(substituted phenyl)methyl) phosphonate **7** via hydrolysis [22]. The α-aminophosphonates **7** were then coupled **3** to provide compounds **8a**–**8j** in satisfactory yields, which was purified by column chromatography over silica gel.

### 2.2. Cytotoxicity against Tumor Cells

Table 1 shows the IC_50_ values (cytotoxicity potency indexes) and the inhibitory rates of cell viability of compounds **8a**–**8j** against HCT-116, human KB and MGC-803 cells in culture. All the compounds were found to be more toxic against human KB cells than HCT-116 and MGC-803 cells. The lead compound **4-MU** exhibited insignificant cytotoxicity with high IC_50_ values, more than 100 μM on HCT-116 cells and more than 50 μM on human KB cells. In comparison, most of the derivatives had much more potent cytotoxicity with significantly lower IC_50_ values on the tumor cells. Compound **8j** was the most effective one against HCT-116 and human KB cells with IC_50_ values being eleven to twelve fold lower than those for the lead compound under the same experimental conditions. Therefore, it is suggested that the introduction of α-aminophosphonates to **4-MU** can obviously improve the antitumor activity of the lead compound. In comparison, activities of compounds against normal cells (Human Umbilical Vein Endothelial Cells, HUVEC) were also examined (Table 1). The IC_50_ values proved that the anti-proliferative activity of some compounds were much higher against cancer cells than normal cells. Compound **8j** exhibited more sensitivity to cancer cells compared to normal cells. Cytotoxicity of the some compounds were much better than 5-fluoro-2,4(1*H*,3*H*)pyrimidinedione (5-Fu) against cancer cells. The IC_50_ values of **8j** against the MGC-803, HCT-116 and human KB cell lines were 14.55 μM, 8.68 μM and 0.08 μM, respectively, whereas those of 5-Fu were 15.32 μM, 10.05 μM, and 1.23 μM, respectively.

**Table 1:** Cytotoxic evaluation under no irradiation of compounds **8a**–**8j**.

Compounds	R^1^	R^2^	IC_50_^a^, μM			
			MGC-803	HUVEC	HCT-116	KB
**2**	-	-	>100	>100	>100	>50
**8a**	H	Ph	>100	>100	>100	>50
**8b**	H	*o*-F-Ph	>100	>100	>100	>50
**8c**	H	*o*-Cl-Ph	79.38 ± 3.82	>100	88.68 ± 1.69	33.4 ± 1.20
**8d**	H	*p*-Cl-Ph	76.85 ± 3.89	>100	86.18 ± 1.86	7.8 ± 5.36
**8e**	H	*o*-Br-Ph	67.49 ± 3.26	>100	90.55 ± 1.09	9.55 ± 0.09
**8f**	H	*m*-Br-Ph	74.16 ± 3.36	>100	25.75 ± 1.21	0.50 ± 2.68
**8g**	H	*p*-Br-Ph	85.63 ± 2.83	>100	88.52 ± 4.62	7.27 ± 6.18
**8h**	H	*o*-OCH_3_-Ph	36.85 ± 1.96	>100	23.68 ± 5.89	0.45 ± 1.03
**8i**	H	2-Na	41.47 ± 2.86	>100	38.90 ± 0.04	0.79 ± 1.70
**8j**	CH_3_	Ph	14.55 ± 1.11	>100	8.68 ± 3.20	0.08 ± 1.12
**5-Fu**			15.32 ± 1.96	56 ± 3.21	10.05 ± 2.20	1.23 ± 1.09

^a^ IC_50_ values are presented as the mean ± SD (standard error of the mean) from three separated experiments.

According to the antitumor activity data (Table 1), the IC_50_ values of the compounds, which have α-aminophosphonates containing bromo-substituted phenyl (R^2^) and hydrogen (R^1^) as side chains, were in the order of **8e** > **8g** > **8f**. It was the same to derivatives with chloro-substituted phenyl (R^2^) and hydrogen (R^1^) (**8c** > **8d**). The IC_50_ values of **8h**, bearing α-aminophosphonate with *ortho*-methoxyl-substituted phenyl (R^2^) and hydrogen (R^1^) as side chain, against the HCT-116 and human KB cell lines were 23.68 μM and 0.45 μM, respectively, whereas those of **8d** and **8i** were 86.18 μM and 7.8 μM, 38.90 μM and 0.79 μM, respectively. Compound **8j**, which has methyl and phenyl as R^1^ and R^2^, respectively, was the most effective one. From the above results, some interesting structure-activity relationships can be disclosed that: (i) the substituted position of the phenyl group of α-aminophosphonate have evident effect on the antitumor activity: meta-substitution > para-substitution > othor-substitution; (ii) the different substituents of the phenyl group of α-aminophosphonate have different effects on the antitumor activity: methoxylgroup > bromine atom > chlorine atom > fluorine atom, an explanation for this phenomenon may be because the differences of their electrical charge cause different lipotropy which can influence diffuse through the plasma membrane; (iii) Compared with hydrogen as R^1^, the methyl as R^2^ shows higher cytotoxicity.

### 2.3. DNA Binding Properties

#### 2.3.1. Fluorescence Emission Titration

**Figure 1:**
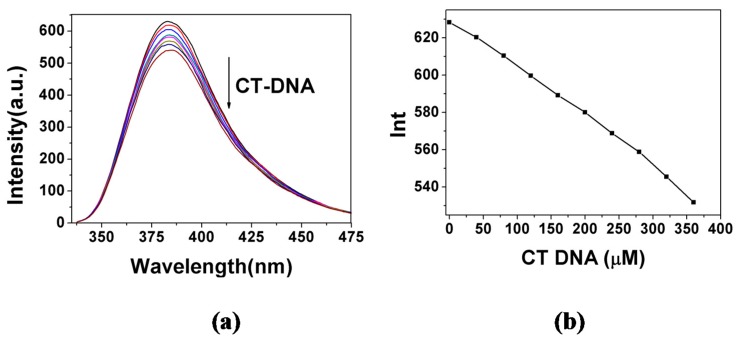
(**a**) Spectrofluorimetric titration of **8j** (2.0 × 10^−3^ M) with CT-DNA of increasing concentration (0–360 μM) in 2.5 mL Tris-HCl buffer (pH = 7.2) at room temperature, ex 325 nm; (**b**) the peak fluorescence intensity in (**a**) at 383 nm *vs.* CT-DNA concentration.

The DNA binding properties of compounds **8a**–**8j** were evaluated based on their affinity or interaction with CT-DNA, measured with fluorescence spectrometric titration [23]. Figure 1a shows the fluorescence spectrometric titration spectra of **8j**, and Figure 1b shows the corresponding peak fluorescence intensity at increasing CT-DNA concentration. The results show that the fluorescence of **8j** could be quenched by DNA, and increasing the concentration of CT-DNA resulted in a decrease in fluorescence intensity of **8j**. The fluorescence quenching of **8j** by CT-DNA indicated that **8j** had a strong interaction with CT-DNA as most of the intercalators did [24]. The titration spectra and peaks for other compounds exhibited the similar trend of changes with the CT-DNA concentration (see Figures S1–S10).

**Figure 2:**
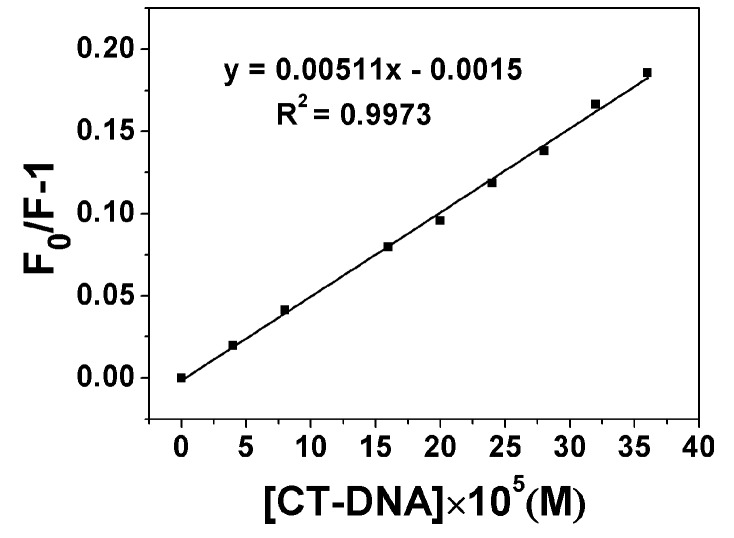
Plot of [DNA] × 10^5^ *vs.* (*F*_0_/*F*-1) of compound **8j**.

The calibration curve for determination of DNA was constructed by adding different proportion of DNA to a 2.0 × 10^−3^ M compound **8j** solution (pH = 7.2 Tris-HCl buffer). The results exhibited good consistency with the Stern-Volmer equation [25]:

(1) $$F_{0}/F\text{ = 1 + }K_{SV}\text{[Q] = 1 + }K_{Q}\tau_{0}\text{[Q]}$$

where *F*_0_ and *F* are the steady-state fluorescence intensities in the absence and presence of the quencher, respectively. [Q] is the concentration of quencher. *K_SV_* is the Stern-Volmer constant and *K_Q_* is the bimolecular quenching rate constant. τ_0_ is the lifetime of the fluorophore in the absence of quencher and for most biomolecules τ_0_ is about 10^−8^ s [26]. The Stern-Volmer constant, *K_SV_*, for compound **8j** has been determined from the plot of *F*_0_/*F*-1 *vs.* [DNA] (see Figure 2) and found to be 5.11 × 10^2^ M^−1^. The Stern-Volmer constants *K_SV_* for other compounds were listed in Table 2. The plots of *F*_0_/*F*-1 *vs.* [DNA] for other compounds were showed in Figures S11–S20.

The Stern-Volmer plot is linear for **8j**, indicating that only one type of quenching process occurs. The Stern-Volmer quenching is largely higher than the maximum collision diffuse quenching constant of the biomolecules (2.0 × 10^10^ L∙mol^−^^1^∙s^−^^1^), indicating that the fluorescence quenching was mainly arisen from static quenching by complex formation instead of dynamic quenching [27]. The Stern-Volmer plots are linear for other compounds, too.

**Figure 3:**
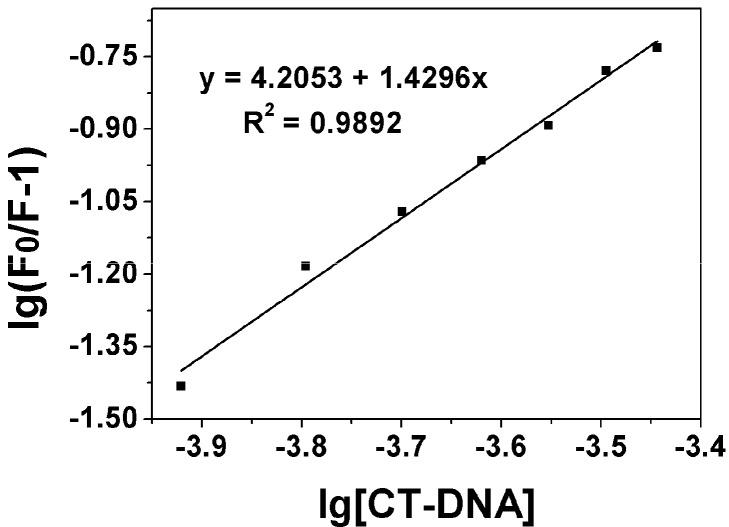
Plot of lg[DNA] *vs.* lg(*F*_0_/*F*-1), *K_b_* = 7.81 × 10^4^ M^−^^1^ of compound **8j**.

When small molecules binding independently to a set of equivalent sites on a macromolecule, the equilibrium between free and bound molecules is given by the equation [28]:

(2) $$\text{lg (}F_{0}/F\text{-1) = lg}K_{b}\text{ + }n\text{lg[Q]}$$

where *K_b_* and *n* are the binding constant and the number of binding sites, respectively, which could be got from the curve of lg(*F*_0_/*F*-1) *vs.* lg[Q]. The plot of lg(*F*_0_/*F*-1) *vs.* lg[DNA] has been shown in Figure 3 for compound **8j** and indicates a simple binding process for the compound. The binding constants *K_b_* and the number of binding sites n of all the derivatives were listed in Table 2. The plots of lg(*F*_0_/*F*-1) *vs.* lg[DNA] for other compounds were showed in Figures S21–S30.

Typical-binding constant between organic compound and DNA usually ranges from 10^4^–10^6^ M^−1^, Table 2 shows that some compounds are actually moderate DNA-intercalators. The relative binding affinities as indicated by the binding constants *K_b_* are in the order of **8h** > **8j** > **8i** > **8f** > **8d** > **8g** > **4-MU** > **8a** > **8e** > **8c** > **8b**. Therefore, more than half of the derivatives had a higher DNA binding constant than their lead compound **2**. This result indicated that introduction of some aminophosphonates side chains could dramatically increase the DNA binding capacity of **4-MU**.

**Table 2:** Stern-Volmer constants and binding data for compounds **8a**–**8j**.

Compound	*K_b_* (M^−1^)	*n*	*K_SV_*(M^−1^)
**2**	4.56 × 10^2^	1.00	4.42 × 10^2^
**8a**	2.44 × 10^2^	0.96	3.4 × 10^2^
**8b**	77.5	0.83	2.99 × 10^2^
**8c**	1.30 × 10^2^	0.83	4.81 × 10^2^
**8d**	5.91 × 10^2^	1.05	4.29 × 10^2^
**8e**	1.37 × 10^2^	0.84	4.75 × 10^2^
**8f**	1.16 × 10^4^	1.42	4.12 × 10^2^
**8g**	5.76 × 10^2^	1.04	4.1 × 10^2^
**8h**	7.38 × 10^5^	1.95	3.57 × 10^2^
**8i**	1.24 × 10^4^	0.96	1.81 × 10^4^
**8j**	1.6 × 10^4^	1.43	5.11 × 10^2^

In most cases, compounds strongly binding to DNA are high cytotoxic agent. Among the derivatives, those with stronger DNA binding affinities, **8f**, **8h**, **8i** and **8j** exhibited lower IC_50_ values against the tumor cells. In contrast, the other compounds exhibited no or low activity, showing low-affinity with DNA. Compound **8h**, having *ortho*-methoxyl-substituted phenyl as R^2^, had the largest DNA binding constant, it may be attributed to the strong binding of DNA molecules through electrostatic interaction and hydrogen bonding by its methoxyl group.

Steady-state competitive binding experiments using **8f**, **8h** and **8j** as quenchers were undertaken to get further proof for the binding of the compounds to DNA. The binding abilities of these compounds to CT-DNA were primarily investigated by competitive binding in which they served as an intercalative binding probe in competition with GelRed. GelRed, which is environmentally safe and ultra-sensitive for DNA staining, is a newly developed DNA intercalator to replace the classic DNA intercalator EB. Furthermore, both GelRed and EB bound with CT-DNA emit characteristic fluorescence at around 590 nm under 350 nm excitation [15].

**Figure 4:**
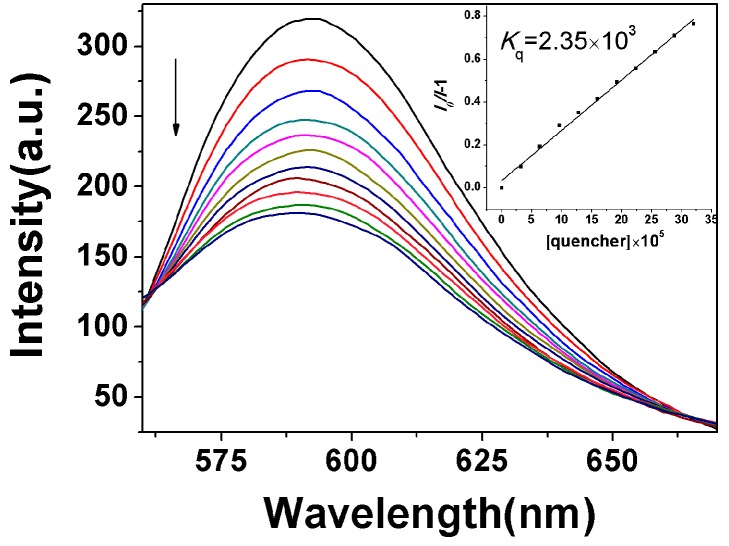
Emission spectra of DNA-GelRed (165 μM), in the presence of 0, 40, 80, 120, 160, 200, 240, 280, 320, 360, 400 and 440 μM of compound **8j**. Arrow indicates the changes in the emission intensity as a function of compound concentration. Inset: Sterne-Volmer plot of the fluorescence titration data corresponding to the compound **8j**.

In competitive binding experiments, GelRed and CT-DNA solutions were pre-incubated for 30min to ensure sufficient interactions. The concentration ratio of GelRed to DNA was set at [GelRed]/[DNA] = 1:10 for sufficient binding sites of DNA for GelRed. The emission spectra of the GelRed-CT-DNA system in the absence and presence of **8j** are shown in Figure 4 (for those of **8f** and **8h**, see Figures S31 and S32, respectively). GelRed-DNA binary solution system gave a characteristic fluorescence emission at around 590 nm when excited at 350 nm, indicating that GelRed molecules intercalated between the adjacent base pairs of DNA and were sufficiently prevented from fluorescence quenching by polar solvent molecules. The addition of compounds **8f**, **8h** or **8j** into the GelRed/CT-DNA binary binding system induced a significant fluorescence quenching of GelRed/CT-DNA. These results strongly suggested the existence of competitive intercalative binding between GelRed and **8f**, **8h** or **8j**, in which compounds **8f**, **8h** or **8j** could displace the GelRed molecules from the DNA neighboring base pairs and there after push the GelRed molecules back into the aqueous solution.

The quenching abilities of compounds **8f**, **8h** and **8j** to GelRed fluorescence can be quantitively estimated by their respective quenching constant, *K*_q_, which was derived from the Stern-Volmer quenching equation. The quenching of GelRed bound to DNA by the test compounds are in good agreement with the linear Sterne-Volmer equation. The values of *K_q_* corresponding to the three compounds **8f**, **8h** and **8j**, obtained by plotting *I*_0_/*I vs.* [Q] , were 1.73 × 10^3^ M^−1^, 2.41 × 10^3^ M^−1^ and 2.35 × 10^3^ M^−1^, respectively. Meanwhile, it can also be confirmed that the intercalative binding mode of the test compounds to DNA was similar to that of GelRed [29]. These values suggested that the compound **8h** exhibited more intensive intercalation to DNA than the compounds **8f** and **8j** which is consistent with the fluorescence spectrometric titration results.

#### 2.3.2. Circular Dichroism Spectra

The circular dichroism (CD) is a useful technique to assess whether the nucleic acids undergo conformational changes as a result of complex formation or changes in environmental conditions [30].

**Figure 5:**
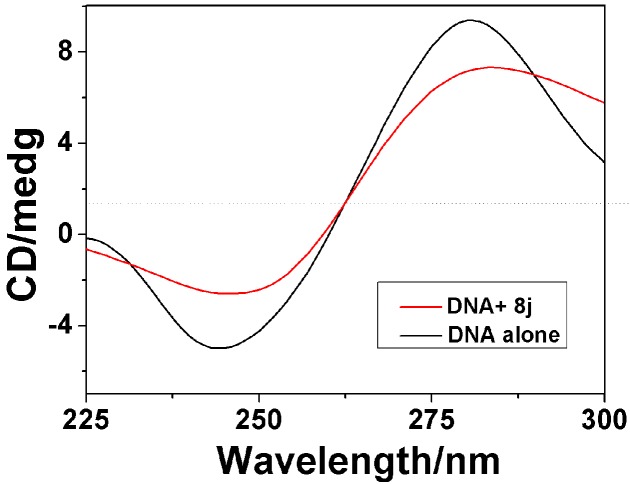
CD spectra of CT-DNA (3 mL solution, 1.5 × 10^−4^ M) in the absence and presence of compound **8j** (1.5 × 10^−5^ M).

As indicated in Figure 5, the CD spectra of CT-DNA (1.5 × 10^−4^ M) showed a positive absorption peak at 281 nm and a negative absorption peak at 247 nm due to π-π base stacking and right-hand helicity, respectively, which is consistent with the characteristic B conformation of DNA [31]. The CD absorption of CT-DNA in the presence of compound **8j** showed a significant decrease in the intensity at the [**8j**]/[DNA] ratio of 1:10. The decrease percentages in the DNA maximal positive and negative absorption by **8j** were of 26.82% and 30.4%, respectively. It suggests that compound **8j** may intercalate between the neighboring base pairs of CT-DNA mainly due to the aromatic planarity of the coumarin, because the decreases in the intensities of both the positive and negative bands can usually be observed in the intercalative binding of small molecules to DNA [32]. The CD absorptional curves of CT-DNA in the presence of compounds **8h** and **8f** were similar to that of compound **8j** (for those of **8f** and **8h**, see Figures S33 and S34, respectively), which suggests the comparable DNA binding properties of all the three compounds due to their same binding mode. The calculated results for the changes on intensities of both the positive and negative CD absorption peak of CT-DNA bound with compounds **8j**, **8h** and **8f** were listed in Table 3.The changes in CD signals of DNA after adding **8j**, **8h** and **8f** were consistent with Scatchard-binding constants, **8h** > **8f** > **8j**, which indicated that DNA-binding ability of **8h** is stronger than those of **8f** and **8j**. These results were consistent with the competitive binding fluorescence measurements of **8j**, **8h** and **8f**. Furthermore, no appreciable red-or blue-shifts in wavelengths were observed and the shape of the CT-DNA absorption curve was unchanged, indicating that there was no significant transformation in CT-DNA secondary structure [33].

**Table 3:** Calculated changes on intensities of both the positive and negative CD absorption peak of CT-DNA bound with Compounds **8j**, **8h** and **8f**.

Compounds	8f	8h	8j
Positive AI decrease(%) ^a^	23.1	34.5	26.82
Negative AI decrease(%) ^a^	26.9	28.5	30.4

^a^ AI: Absorption Intensity.

### 2.4. Apoptosis and Cell Cycle Analysis

**Figure 6:**
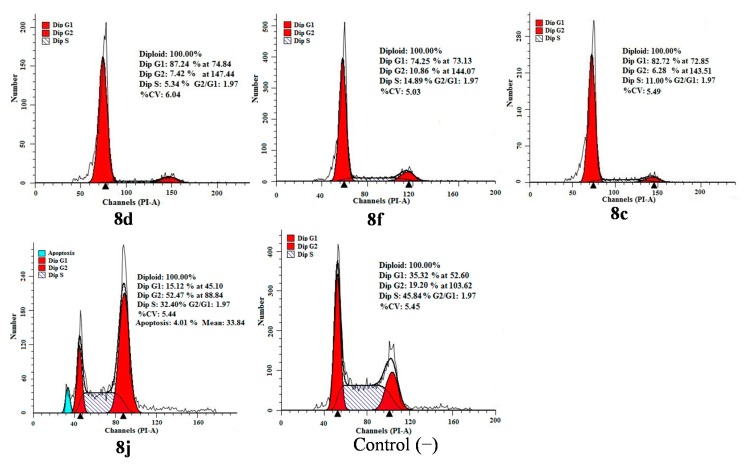
Inhibition of cell cycle progress in HCT-116 cells treated with compounds **8d**, **8f**, **8c** and **8j** for 48 h. Cells were fixed with ethanol and stained with PI. Cell cycle distribution was analyzed by flow cytometry. First peak represents sub-G1 peak, which was taken as the fraction of the apoptotic cell population.

Flow-cytometric analysis further confirmed tumor cell apoptosis as shown in Figure 6. Cytometric profiles of the PI-stained DNA showed cell cycle arrest of HCT-116 cells treated with compounds **8c**, **8d**, **8f** and **8j** for 48 h at IC_50_ concentrations. Compared to the control (untreated cells), changes in the cell cycle distribution of treated HCT-116 cells were evident. G1-phase populations of 87.24% for **8d**, 74.25% for **8f** and 82.72% for **8c** were observed compared with a G1-phase population of 35.32% for untreated cells. For the compound **8j**, the sub-G1 peak and the accumulation of cells in the G2 phase accompanying reduction in the G1 phase were observed after the cells were exposed to **8j**.

### 2.5. Release of Cytochrome c and Activation of Caspases were Involved in the Apoptosis Induced by **8j**

**Figure 7:**
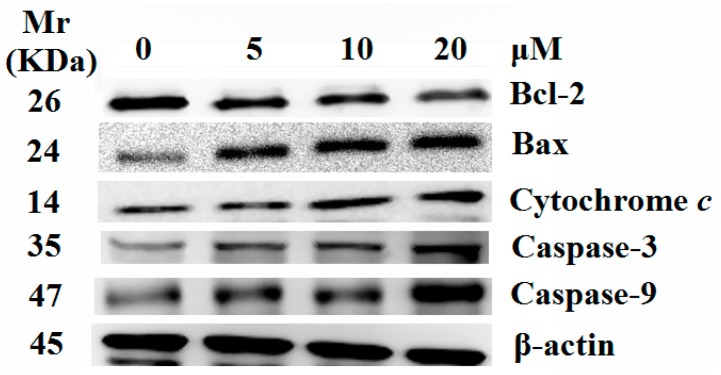
Effect of compound **8j** on cytochrome *c* release and levels of Bcl-2, Bax, Caspase-9 and Caspase-3. Equal amount of protein was loaded on SDS-PAGE gel for western blot analysis as described in experimental section. β-actin was used as an internal control.

To confirm the molecular mechanisms involved in the observed apoptosis alterations, we investigated the effects of compound **8j** on the expression of proteins important for mitochondria mediated apoptosis. Apoptosis occurs through two different pathways, that is the intrinsic and extrinsic pathways. Intrinsic pathway involves mitochondria that play a pivotal role in apoptosis, whereas extrinsic pathway is mediated by cell death receptors such as Fas, TNF α after receiving the death signal. It is well-known that the intrinsic pathway is regulated by the Bcl-2 family of pro- and anti- apoptotic proteins, which stimulate the permeabilization of the mitochondrial outer membrane and cytochrome *c* released into the cytosol, promoting in the activation of the caspase cascade and the induction of apoptotic cell death. The effects of compound **8j** on the constitutive levels of Bax, Bcl-2 and cytochrome *c* in HCT-116 cells are given in Figure 7. In comparison with the control cells, **8j** induced a significant increase in the levels of Bax and a decrease in the expression of Bcl-2, in a dose-dependent manner. Compound **8j** treatment caused an accumulation of cytochrome *c* in the cytosol, most probably due to the release of mitochondrial cytochrome *c.* These results indicated an involvement of caspases in the apoptotic process downstream of mitochondria. Then, the roles of important caspases (caspase-9 and caspase-3) were investigated. As Figure 7 shown, the treatment of HCT-116 cells with **8j** caused a marked increase in the levels of caspase-3 and caspase-9 proteins compared to the control. These results revealed an involvement of caspases in the intrinsic apoptotic process downstream of mitochondria. Thus, compound **8j** induced HCT-116 cells apoptosis might decrease the activation of Bcl-2 and stimulate its downstream proteins which are associated with the mitochondria-dependent apoptotic pathway.

## 3. Experimental Section 

### 3.1. Chemistry

All chemicals (reagent grade) used were commercially available. NMR spectra were measured on a BRUKER AVANCE AV500 spectrometer (Bruker Corporation, Karlsruhe, Germany) using TMS as the internal standard. The mass spectra were obtained on a BRUKER ESQUIRE HCT spectrometer (Bruker Corporation). Melting points were determined using an X-4 apparatus (Huier Corporation, Zhejiang, China) and were uncorrected. The elemental analyses (C, H and O) were carried out on a Perkin Elmer Series II CHNS/O 2400 elemental analyzer (Perkin Elmer Corporation, Waltham, MA, USA).

All the chemical reagents and the solvents were analytical grade. Calf thymus DNA (CT-DNA) was purchased from Sigma-Aldrich (Saint Louis, MO, USA). They were all used as received without further purification unless noted specifically.

#### 3.1.1. 7-Hydroxy-4-methyl-2*H*-chromen-2-one (**2**)

A mixture of phenols (1 mmol) and ethyl acetoacetate (1 mmol) was ground with sodium bisulfate (2 mmol) in a mortar by pestle for 10 min when a color change of the reaction mixture took place. The reaction mixture was kept at room temperature for about 20–80 min. The completion of the reaction was checked by TLC (silica gel using solvent petroleum ether:acetone, 2:1). The reaction mixture was diluted with ice-cold water. The solid that separated out was filtered at vacuum, washed with water, and recrystallized from ethanol to give pure **4-MU** in 96% yield. White acicular crystal. m.p. 185–187 °C. ^1^H-NMR (500 MHz, CDCl_3_) δ 8.45 (dd, *J* = 9.6, 3.4 Hz, 1H), 6.75 (dd, *J* = 8.8, 2.5 Hz, 1H), 6.70 (d, *J* = 2.5 Hz, 1H), 6.09 (d, *J* = 1.1 Hz, 1H), 2.34 (d, *J* = 1.1 Hz, 3H).

#### 3.1.2. 3-(4-Methyl-2-oxo-2*H*-chromen-7-yloxy)propanoic Acid (**3**)

In 500 mL of RBF, provide with reflux condenser **4-MU** (0.01 mol), 3-bromopropionic acid (0.01 mol) and K_2_CO_3_ (0.03 mol) in 25 mL of distilled acetone. Then resultant solution was refluxed in water bath. After 1 h, pH of the solution had dropped to 7, and further 1 g of K_2_CO_3_ was added. Refluxing was continued for a 12 h. The hot solution was acidified with dilute hydrochloric acid. The product was extracted with diethyl ether. Ether layer was washed with 50 mL of saturated solution of sodium bicarbonate. The aqueous layer was acidified with HCl acid. White colored solid was filtered off, washed with water, dried and recrystallized by hot water to get the desired white compounds (**3**), yield 65%. White solid. m.p. 140–142 °C.^1^H-NMR (500 MHz, CDCl_3_) δ 7.42 (d, *J* = 8.8 Hz, 1H), 6.75 (dd, *J* = 8.8, 2.5 Hz, 1H), 6.70 (d, *J* = 2.5 Hz, 1H), 6.09 (d, *J* = 1.1 Hz, 1H), 4.27 (t, *J* = 6.3 Hz, 2H), 2.76 (t, *J* = 6.3 Hz, 2H), 2.34 (d, *J* = 1.1 Hz, 3H). ^13^C-NMR (125 MHz, CDCl_3_) δ (ppm) 172.02, 160.05, 159.26, 155.20, 150.18, 127.30, 112.31, 112.26, 110.96, 102.17, 65.67, 34.76, 20.28. Anal. for C_13_H_12_O_5_: calcd C, 62.90%; H, 4.87%; O, 32.23%; found C, 62.88%; H, 4.86%; O, 32.26%.

#### 3.1.3. General Procedure for the Preparation of the *O*-Substituted Coumarin Derivatives **8a**–**8j**

A suspension of aromatic aldehyde (aromatic ketone) (10 mmol) and ammonium acetate (11 mmol) was stirred for 6 h at reflux. The reaction mixture was filtered to give the white precipitate **5**, to which the diethyl phosphite (5 mmol) was added and the resulting solution was stirred for 3 h at 70 °C. After that, hydrochloric acid (4 mmol) in 30 mL ether was added to the reaction mixture, which was stirred for 2 h at 0 °C to give the precipitate **6**. The precipitate was filtered and washed with ether (15 mL), and then it was added to 10 mL sodium hydroxide solution (15%) and stirred for 30 min at room temperature. Extraction with dichloromethane (3 × 30 mL) and evaporation of the solvent gave the oils of **7**, which was purified by column chromatography on silica gel with ethyl acetate. Compound **3** (0.6 mmol), α-aminophosphonates (0.6 mmol), HOBT (0.6 mmol), and EDAC (1.0 mmol) in dry DMSO (10 mL) were stirred in ice bath. After 10 min, the reaction mixture was stirred for 6–8 h at room temperature (TLC detection). The mixture was washed by water and ethyl acetate (water/ethyl acetate = 1:2, *v*/*v*) for three times. The solvent was removed under reduced pressure. The residue was purified by chromatography on silica gel with a solution of petroleum ether and ethyl acetate (water/ethyl acetate = 1:2, *v*/*v*) as eluent to give desired product.

*Diethyl(3-(4-methyl-2-oxo-2H-chromen-7-yloxy)propanamido)(phenyl)methylphosphonate* (**8a**). Yield 81%. White solid. m.p. 126–128 °C. ^1^H-NMR (500 MHz, CDCl_3_) δ (ppm) 7.53–7.49 (m, 2H), 7.42 (d, *J* = 8.8 Hz, 1H), 7.29 (qd, *J* = 7.9, 1.3 Hz, 3H), 6.75 (dd, *J* = 8.8, 2.5 Hz, 1H), 6.70 (d, *J* = 2.5 Hz, 1H), 6.09 (d, *J* = 1.1 Hz, 1H), 5.61 (dd, *J* = 21.0, 9.7 Hz, 1H), 4.30–4.23 (m, 2H), 4.18–3.59 (m, 4H), 2.76 (t, *J* = 6.3 Hz, 2H), 2.34 (d, *J* = 1.1 Hz, 3H), 1.31 (t, *J* = 7.1 Hz, 3H), 1.05 (t, *J* = 7.1 Hz, 3H). ^13^C-NMR (125 MHz, CDCl_3_) δ (ppm) 170.01, 164.05, 161.33, 161.01, 155.21, 150.58, 128.65, 128.16, 127.24, 126.51, 114.32, 112.53, 112.41, 102.26, 64.98, 64.29, 64.18, 49.23, 36.26, 19.26, 16.84, 16.80. ^31^P-NMR (202MHz, CDCl_3_) δ (ppm) 21.49 (s). HRMS for C_24_H_29_NO_7_P ([M + H]^+^): calcd 474.16816: found 474.16656.

*Diethyl(3-(4-methyl-2-oxo-2H-chromen-7-yloxy)propanamido)(2-fluorophenyl)methylphospho-nate* (**8b**). Yield 85%. White solid. m.p. 130–132 °C. ^1^H-NMR (500 MHz, CDCl_3_) δ (ppm) 8.30 (d, *J* = 7.4 Hz, 1H), 7.46 (ddd, *J* = 8.6, 5.1, 2.0 Hz, 2H), 7.41 (d, *J* = 8.8 Hz, 1H), 6.97 (t, *J* = 8.6 Hz, 2H), 6.73 (dd, *J* = 8.8, 2.5 Hz, 1H), 6.70 (d, *J* = 2.4 Hz, 1H), 6.08 (d, *J* = 1.1 Hz, 1H), 5.56 (dd, *J* = 21.0, 9.6 Hz, 1H), 4.25 (ddd, *J* = 15.5, 9.3, 3.1 Hz, 2H), 4.18–3.65 (m, 4H), 2.74 (t, *J* = 6.2 Hz, 2H), 2.34 (d, *J* = 1.1 Hz, 3H), 1.29 (t, *J* = 7.1 Hz, 3H), 1.07 (t, *J* = 7.1 Hz, 3H). ^13^C-NMR (125 MHz, CDCl_3_ ) δ (ppm) 170.16, 161.90, 161.27, 155.58, 152.89, 142.28, 134.16, 131.10, 129.79, 127.66, 127.27, 126.16, 112.61, 112.46, 110.98, 102.24, 65.06, 64.16, 64.08, 45.99, 35.68, 20.20, 16.96, 16.73. ^31^P-NMR (202 MHz, CDCl_3_) δ (ppm) 21.20 (d, *J* = 4.3 Hz). HRMS for C_24_H_28_NO_7_ FP ([M + H]^+^): calcd 492.15874: found 492.15732.

*Diethyl(3-(4-methyl-2-oxo-2H-chromen-7-yloxy)propanamido)(2-chlorophenyl)methylphospho-nate* (**8c**). Yield 82%. White solid. m.p. 115–117 °C. ^1^H-NMR (500-MHz, CDCl_3_) δ (ppm) 8.01 (s, 1H), 7.47 (d, *J* = 8.8 Hz, 1H), 7.42–7.21 (m, 4H), 6.81 (dd, *J* = 8.8, 2.5 Hz, 1H), 6.75 (d, *J* = 2.4 Hz, 1H), 6.22–6.16 (m, 1H), 6.14 (s, 1H), 4.36–4.30 (m, 2H), 4.30–3.64 (m,4H), 2.79 (td, *J* = 6.1, 2.6 Hz, 2H), 2.39 (d, *J* = 0.8 Hz, 3H), 1.36 (t, *J* = 7.1 Hz, 3H), 1.06 (t, *J* = 7.0 Hz, 3H). ^13^C-NMR (125 MHz, CDCl_3_ ) δ (ppm) 170.04, 161.95, 161.76, 155.65, 153.02, 142.63, 134.08, 130.27, 129.89, 127.57, 127.10, 126.12, 112.69, 112.59, 111.10, 102.34, 64.21, 64.04, 63.98, 47.89, 36.35, 19.20, 17.00, 16.62. ^31^P-NMR (202 MHz, CDCl_3_) δ (ppm) 20.72 (s). HRMS for C_24_H_28_NO_7_PCl ([M + H]^+^):calcd 508.12919; found 508.12781.

*Diethyl(3-(4-methyl-2-oxo-2H-chromen-7-yloxy)propanamido)(4-chlorophenyl)methylphospho-nate* (**8d**). Yield 85%. White solid. m.p. 110–112 °C. ^1^H-NMR (500 MHz, CDCl_3_) δ (ppm) 8.21 (s, 1H), 7.46 (dd, *J* = 8.5, 2.2 Hz, 1H), 7.42 (d, *J* = 8.3 Hz, 2H), 7.29 (dd, *J* = 8.3, 1.6 Hz, 2H), 6.78 (d, *J* = 2.5 Hz, 1H), 6.76 (s, 1H), 6.13 (d, *J* = 1.0 Hz, 1H), 5.58 (dd, *J* = 21.3, 9.5 Hz, 1H), 4.40–4.23 (m, 2H), 4.21–3.72 (m, 4H), 2.78 (t, *J* = 6.0 Hz, 2H), 2.38 (d, *J* = 1.0 Hz, 3H), 1.32 (t, *J* = 7.0 Hz, 3H), 1.12 (dd, *J* = 7.7, 6.4 Hz, 3H). ^13^C-NMR (125 MHz, CDCl_3_) δ (ppm) 170.21, 164.45, 161.83, 161.67, 155.51, 150.98, 133.86, 129.95, 129.16, 126.01, 114.23, 112.64, 112.51, 102.05, 64.95, 64.19, 64.13, 49.22, 36.22, 19.07, 16.87, 16.83. ^31^P-NMR (202 MHz, CDCl_3_) δ (ppm) 21.21 (s). HRMS for C_24_H_28_NO_7_ PCl ([M + H]^+^): calcd 508.12919; found 508.12775.

*Diethyl(3-(4-methyl-2-oxo-2H-chromen-7-yloxy)propanamido)(2-bromophenyl)methylphospho-nate* (**8e**). Yield 87%. White solid. m.p. 160–162 °C. ^1^H-NMR (500 MHz, CDCl_3_) δ (ppm) 8.67 (s, 1H), 7.75 (d, *J* = 7.8 Hz, 1H), 7.53 (d, *J* = 8.0 Hz, 1H), 7.40 (dd, *J* = 8.8, 1.2 Hz, 1H), 7.27 (dd, *J* = 12.7, 5.2 Hz, 1H), 7.12 (dd, *J* = 11.0, 4.3 Hz, 1H), 6.73 (dd, *J* = 8.8, 2.4 Hz, 1H), 6.67 (d, *J* = 2.3 Hz, 1H), 6.20 (dd, *J* = 21.0, 9.3 Hz, 1H), 6.07 (s, 1H), 4.34–4.23 (m, 2H), 4.24–3.58 (m, 4H), 2.77 (q, *J* = 6.1 Hz, 2H), 2.33 (s, 3H), 1.34 (t, *J* = 7.1 Hz, 3H), 1.02 (t, *J* = 7.1 Hz, 3H). ^13^C-NMR (125 MHz, CDCl_3_) δ (ppm) 170.15, 162.00, 161.86, 155.56, 153.58, 147.69, 132.68, 130.34, 129.80, 128.00, 127.91, 126.35, 112.49, 112.21, 111.12, 102.43, 64.20, 64.06, 63.88, 47.86, 36.34, 19.25, 16.50, 16.39. ^31^P-NMR (202MHz, CDCl_3_) δ (ppm) 20.85 (s). HRMS for C_24_H_28_NO_7_PBr ([M + H]^+^): calcd 552.07868; found 552.07666.

*Diethyl(3-(4-methyl-2-oxo-2H-chromen-7-yloxy)propanamido)(3-bromophenyl)methylphospho-nate* (**8f**). Yield 83%. White solid. m.p. 171–172 °C. ^1^H-NMR (500 MHz, CDCl_3_) δ (ppm) 8.36 (d, *J* = 5.7 Hz, 1H), 7.67 (d, *J* = 1.5 Hz, 1H), 7.44 (dd, *J* = 15.1, 8.4 Hz, 2H), 7.20 (t, *J* = 7.8 Hz, 1H), 6.79 (dd, *J* = 8.8, 2.4 Hz, 1H), 6.74 (d, *J* = 2.4 Hz, 1H), 6.13 (d, *J* = 0.7 Hz, 1H), 5.58 (dd, *J* = 21.3, 9.5 Hz, 1H), 4.30 (dq, *J* = 9.3, 6.4 Hz, 2H), 4.26–3.73 (m, 4H), 2.79 (t, *J* = 6.1 Hz, 2H), 2.38 (d, *J* = 0.5 Hz, 3H), 1.33 (t, *J* = 7.1 Hz, 3H), 1.12 (t, *J* = 7.1 Hz, 3H). ^13^C-NMR (125 MHz, CDCl_3_ ) δ (ppm) 170.24, 161.84, 161.62, 155.52, 152.93, 137.99, 131.50, 131.45, 127.43, 127.39, 126.01, 123.01, 114.22, 112.60, 112.52, 102.10, 64.99, 64.05, 63.93, 49.42, 36.17, 19.08, 16.88, 16.58. ^31^P-NMR (202 MHz, CDCl_3_) δ (ppm) 21.71 (s). HRMS for C_24_H_28_NO_7_PBr ([M + H]^+^): calcd 552.07868; found 552.07642.

*Diethyl(3-(4-methyl-2-oxo-2H-chromen-7-yloxy)propanamido)(4-bromophenyl)methylphosph-onate* (**8g**). Yield 87%. White solid. m.p. 110.8–112.8 °C. ^1^H-NMR (500 MHz, CDCl_3_) δ (ppm) 8.23 (dd, *J* = 9.5, 4.1 Hz, 1H), 7.43 (d, *J* = 8.1 Hz, 2H), 7.41 (s, 1H), 7.33 (dd, *J* = 8.5, 2.0 Hz, 2H), 6.75 dd, *J* = 8.8, 2.4 Hz, 1H), 6.73 (d, *J* = 2.3 Hz, 1H), 6.10 (d, *J* = 1.2 Hz, 1H), 5.54 (dd, *J* = 21.3, 9.5 Hz, 1H), 4.32–4.21 (m, 2H), 4.19–3.70 (m, 4H), 2.75 (t, *J* = 6.1 Hz, 2H), 2.35 (d, *J* = 1.2 Hz, 3H), 1.29 (t, *J* = 7.1 Hz, 3H), 1.09 (t, *J* = 7.1 Hz, 3H). ^13^C-NMR (125MHz, CDCl_3_) δ (ppm) 170.26, 164.49, 161.86, 161.76, 155.67, 150.89, 132.96, 129.98, 129.27, 126.12, 114.36, 112.50, 112.26, 102.06, 64.39, 64.16, 64.12, 47.96, 365.20, 20.12, 16.89, 16.85. ^31^P-NMR (202 MHz, CDCl_3_) δ (ppm) 20.87 (s). HRMS for C_24_H_28_NO_7_PBr ([M + H]^+^): calcd 552.07868; found 552.07629.

*Diethyl(3-(4-methyl-2-oxo-2H-chromen-7-yloxy)propanamido)(2-methoxyphenyl)methylphos-phonate* (**8h**). Yield 81%. White solid. m.p. 128.6–130.6 °C. ^1^H-NMR (500 MHz, CDCl_3_) δ (ppm) 8.08 (dd, *J* = 9.7, 2.9 Hz, 1H), 7.57–7.45 (m, 1H), 7.39 (d, *J* = 8.8 Hz, 1H), 7.24–7.18 (m, 1H), 6.91–6.81 (m, 2H), 6.74 (dd, *J* = 8.8, 2.5 Hz, 1H), 6.68 (d, *J* = 2.5 Hz, 1H), 6.14–6.08 (m, 1H), 6.06 (d, *J* = 1.2 Hz, 1H), 4.30–4.20 (m, 2H), 4.19–3.70 (m, 4H),3.82 (s, 3H), 2.73 (dd, *J* = 10.4, 6.1 Hz, 2H), 2.32 (d, *J* = 1.0 Hz, 3H), 1.28 (t, *J* = 7.1 Hz, 3H), 0.98 (t, *J* = 7.1 Hz, 3H). ^13^C-NMR (125 MHz, CDCl_3_ ) δ (ppm) 170.11, 162.16, 161.86, 155.67, 153.21, 142.47, 134.15, 130.57, 129.98, 128.01, 127.78, 126.42, 112.88, 112.69, 111.86, 102.59, 65.19, 64.25, 64.19, 53.56, 46.90, 35.36, 19.27, 16.70, 16.62. ^31^P-NMR (202 MHz, CDCl_3_) δ (ppm) 22.05 (s). HRMS for C_25_H_31_NO_8_ P ([M + H]^+^): calcd 504.17873; found 504.17688.

*Diethyl(3-(4-methyl-2-oxo-2H-chromen-7-yloxy)propanamido)(naphthalen-2-yl)methylphosph-onate* (**8i**). Yield 82%. White solid. m.p. 177.6–179.6 °C. ^1^H-NMR (500 MHz, CDCl_3_) δ (ppm) 8.46 (s, 1H), 7.98 (s, 1H), 7.79–7.71 (m, 3H), 7.63 (dd, *J* = 8.5, 1.4 Hz, 1H), 7.47–7.39 (m, 2H), 7.32–7.27 (m, 1H), 6.67 (d, *J* = 1.3 Hz, 1H), 6.65 (t, *J* = 1.9 Hz, 1H), 6.07 (s, 1H), 5.80 (dd, *J* = 21.0, 9.6 Hz, 1H), 4.27–3.59 (m, 6H) 2.76 (t, *J* = 6.3 Hz, 2H), 2.29 (s, 3H), 1.34 (t, *J* = 7.1 Hz, 3H), 1.02 (t, *J* = 7.1 Hz, 3H). ^13^C-NMR (125 MHz, CDCl_3_ ) δ (ppm) 170.32, 160.89, 159.76, 154.65, 152.23, 136.67, 133.66, 131.82, 129.97, 128.46, 127.76, 127.45, 127.16, 126.69, 126.33, 125.29, 114.62, 113.60, 112.04, 106.58, 64.67, 63.81, 63.65, 47.67, 35.69, 19.98, 16.36, 16.29. ^31^P-NMR (202 MHz, CDCl_3_) δ (ppm) 21.44 (s). HRMS for C_28_H_31_NO_7_P ([M + H]^+^): calcd 524.18381; found 524.18213.

*Diethyl 1-(3-(4-methyl-2-oxo-2H-chromen-7-yloxy)propanamido)-1-phenylethylphosphonate* (**8j**). Yield 88%. White solid. m.p. 83.6–85.6 °C. ^1^H-NMR (500 MHz, CDCl_3_) δ (ppm) 8.45 (dd, *J* = 9.6, 3.4 Hz, 1H), 7.62-7.45 (m, 2H), 7.42 (d, *J* = 8.8 Hz, 1H), 7.29 (dd, *J* = 7.9, 4.1 Hz, 3H), 6.75 (dd, *J* = 8.8, 2.5 Hz, 1H), 6.70 (d, *J* = 2.5 Hz, 1H), 6.09 (d, *J* = 1.1 Hz, 1H), 4.34–4.22 (m, 2H), 4.22–3.67 (m, 4H), 2.78 (td, *J* = 6.2, 2.7 Hz, 2H), 2.34 (d, *J* = 1.0 Hz, 3H), 2.10 (d, *J* = 16.1 Hz, 1H), 1.31 (t, *J* = 7.1 Hz, 3H), 1.09 (t, *J* = 7.1 Hz, 3H). ^13^C-NMR (125 MHz, CDCl_3_ ) δ (ppm) 170.28, 160.75, 159.80, 155.26, 152.12, 137.36, 128.87, 128.67, 127.86, 126.96, 113.86, 112.92, 112.58, 104.01, 65.53, 63.72, 63.56, 58.51, 36.39, 21.32, 18.36, 16.20, 16.11. ^31^P-NMR (202 MHz, CDCl_3_) δ (ppm) 21.33 (s). HRMS for C_25_H_31_NO_7_P ([M + H]^+^): calcd 488.18381; found 488.18231.

### 3.2. Biological Assays

#### 3.2.1. Cytotoxicityassay *in Vitro*

*Cell Lines.* The following *in vitro* human cancer cell lines were used: HCT-116 (Human colorectal cells), human KB (human nasopharyngeal carcinoma cells), MGC-803 (human lung adenocarcinoma cell line), HUVEC (Human Umbilical Vein Endothelial Cells). The cell lines (HCT-116, KB, MGC-803 and HUVEC) were purchased from the Cell Resource Center of Shanghai Institutes for Biological Sciences, the Academy of Sciences of China.

*Cell Culture.* HCT-116, human KB, MGC-803 and HUVEC cells were cultured in Dulbecco Modified Eagle Medium (DMEM; HyClone, Los Angeles, CA, USA), containing 4.0 mM L-Glutamine and 4500 mg/L Glucose, supplemented with 10% (*v*/*v*) foetalbovine serum (FBS; HyClone). The cell culturemedia was supplemented with penicillin/streptomycin at 100 Units/mL as adherent monolayers. Cell cultures were kept in a humidified incubator with 5% CO_2_ at 37 °C. Stock solutions were prepared in dimethyl sulfoxide (DMSO) and further dilutions were made with fresh culture medium. The concentration of DMSO in the final culture medium was 1%, which had no effect on the cell viability.

*MTT Assay.* Chemosensitivity was assessed using 3-(4,5-dimethylthiazol-2-yl)-2,5-diphenyl tetrazolium bromide (MTT) assay. Briefly, exponentially growing HCT-116 (3000–4000 cells/well), human KB (2000–3000 cells/well) and MGC 803 (2000–3000 cells/well) were seeded into 96-well plates and treated with indicated concentrations of samples for 48 h, and then 10 mL of MTT (10 mg/mL) was added. After incubation for 4 h at 37 °C, the purple formazan crystals (*i.e*., a reduced form of MTT) generated from viable cells were dissolved by adding 100 μL DMSO in each well. The plates were swirled gently for 10 min to dissolve the precipitate, and quantified by measuring the optical density (OD) of the plates at a wavelength of 490 nm on plate reader (TECAN infinite M1000). Each concentration was repeated in three wells and the same experimental conditions were provided for all compounds and MTT analysis was repeated three times for each cell line.

#### 3.2.2. Apoptosis and Cell Cycle Analysis

Apoptosis was quantified by assessing the fraction of cells with sub-G1 DNA content by flow cytometry. The cells lines were treated with indicated concentrations of compounds **4c**, **4d**, **4f** and **4j**. After incubated for 48 h, cells were washed twice with ice-cold PBS, fixed and permeabilized with ice-cold 70% ethanol at −20 °C overnight. The cells were treated with 100 μg/mL RNase A at 37 °C for 30 min after washed with ice-cold PBS, and finally stained with 1 mg/mL propidium iodide (PI) in the dark at 4 °C for 30 min. Analysis was performed with the system software (Cell Quest; BD Biosciences, Franklin Lakes, NJ, USA).

### 3.3. Spectrofluorimetric Titration and DNA Binding Assay

All fluorescence experiments were carried out on a Perkin-Elmer LS50B luminescence spectrometer. Fluorescence spectra were measured at rt using quartz cells of 1cm path. To the solutions of the drugs (2.0 × 10^−3^ M) in 2.5 mL Tris-HCl buffer (pH = 7.2) were added aliquots of CT-DNA (2.0 × 10^−3^ M) solution containing drugs (2.0 × 10^−3^ M) in 2.5 mL Tris-HCl buffer (pH = 7.2). This operation ensured that the concentration of CT-DNA increased gradually from 0 to 360 M, while the concentrations of drugs were kept constant. The mixing was achieved by stirring for 10min. Then, the corresponding fluorescence spectra were measured (ex 325 nm, ex/em 1.5 nm/1.5 nm). Binding constants were derived from the modified.

A solution containing 2 × 10^−4^ M DNA and 2 × 10^−5^ M GelRed ([DNA]/[GelRed] = 10:1) was prepared for GelRed-DNA competitive binding studies. Fluorescence emission spectra were recorded under slit width as 10 nm/10 nm for ex/em, respectively. The quenching constant for comparing the efficiency of fluorescence quenching, *i.e.*, *K_q_*, of each compound was obtained by the linear fit of plotting *I*_0_/*I vs.* [Q], according to the classic Stern-Volmer equation: *I*_0_/*I* = 1 + *K_q_* × [Q] [34], where *I*_0_ and *I* are the peak emission intensity of the GelRed-DNA system in the absence and presence of each compound as quencher, and [Q] is the concentration of quencher. In the fluorescence polarization experiment, each sample was pre-incubated for 40 min before the fluorescence polarization was recorded under the condition of 595 nm emitting wavelength with 350 nm exciting wavelength, and slit width was set as 5 nm/5 nm for ex/em, respectively.

CD absorption spectra of DNA were measured in TBS at a 100 nm/min scan rate in the wavelength range from 200 to 500 nm, with 1.5 × 10^−4^ M DNA in the absence and presence of each compound of 1.5 × 10^−5^ M, respectively. The CD signal of TBS was taken as the background and subtracted from the spectra. All the spectroscopic experiments were performed at 25 °C.

### 3.4. Statistics

The data were processed by the Student’s *t*-test with the significance level *p* ≥ 0.05 using SPSS (IBM, Armonk, New York, NY, USA).

## 4. Conclusions

In summary, a series of novel coumarin-containing aminophosphonates derivatives were synthesized and the cytotoxicity of these derivatives against different cancer cell lines was determined. The introduction of aminophosphonates side chains is an effective way to improve the antitumor activity of **4-MU**, which may be associated with their DNA binding capacity. Compound **8j** exhibited best potency against HCT-116 and KB cells, but low cytotoxicity towards normal human cell. Importantly, it had excellent cell selectivity in HCT-116 and human KB cells. In addition, it was found that **8c**, **8d**, **8f** and **8j** could disturb the cell cycle in HCT-116cells. The results from western-blot analysis provided evidence for an apoptotic cell death, which may be through up-regulating the caspase-3, caspase-9 and cytochrome *c* signaling pathway. DNA-binding experiments suggested that some novel DNA-intercalators were developed based on coumarin-APA system. This research may provide some new suggestions for the design of novel antitumor agent based on coumarin.

## Supplementary Materials

Supplementary materials can be accessed at: http://www.mdpi.com/1420-3049/20/08/14791/s1.
