# Supplementary material for: Novel Coumarin-Containing Aminophosphonatesas Antitumor Agent: Synthesis, Cytotoxicity, DNA-Binding and Apoptosis Evaluation
Source: Molecules. 2015 Aug 13;20(8):14791–809. doi: 10.3390/molecules200814791 (PMC6331810; doi:10.3390/molecules200814791)
Supplement: Supplementary file 1 [file molecules-20-14791-s001.pdf]

# Supplementary Materials

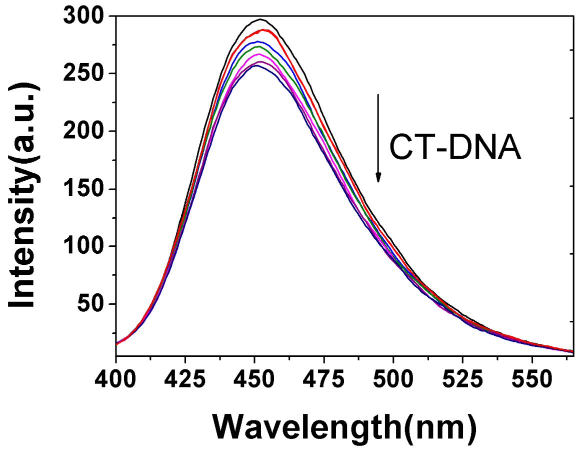


**Figure S1.** Spectrofluorimetric titration of **2** ($2.0 \times 10^{-3}$ M) with CT DNA of increasing volume (0–450 μL, only 0, 100, 200, 250, 350, 400 and 450 μL are shown for clarity) in 2.5 mL Tris-HCl buffer (pH 7.2) at room temperature, ex 325 nm.

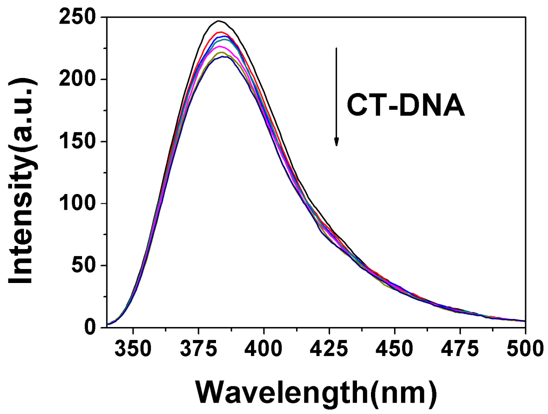


**Figure S2.** Spectrofluorimetric titration of **8a** ($2.0 \times 10^{-3}$ M) with CT DNA of increasing volume (0–450 μL, only 0, 100, 200, 250, 350, 400 and 450 μL are shown for clarity) are shown for clarity in 2.5 mL Tris-HCl buffer (pH 7.2) at roomtemperature, ex 325 nm.

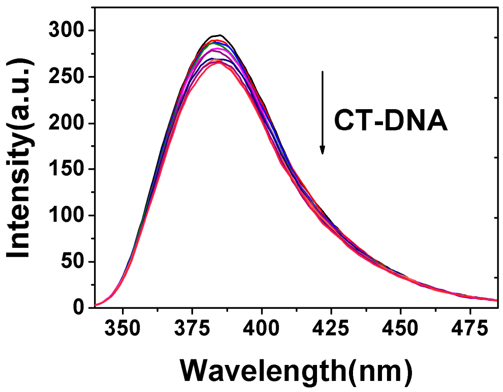


**Figure S3.** Spectrofluorimetric titration of **4b** ($2.0 \times 10^{-3}$ M) with CT DNA of increasing volume (0–450 μL, only 0, 50, 100, 150, 200, 250, 350, 400 and 450 μL are shown for clarity) in 2.5 mL Tris-HCl buffer (pH 7.2) at room temperature, ex 325 nm.

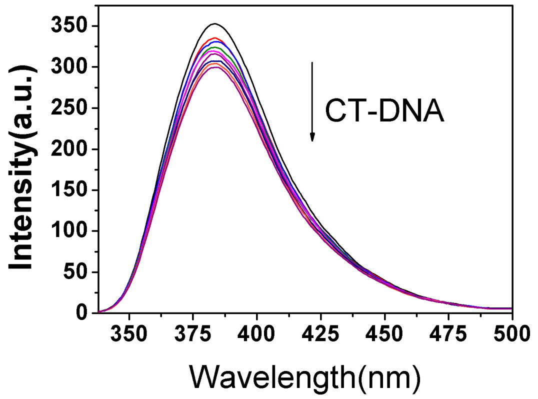


**Figure S4.** Spectrofluorimetric titration of **4c** ($2.0 \times 10^{-3}$ M) with CT DNA of increasing volume (0–450 μL, only 0, 100, 150, 200, 250, 350, 400 and 450 μL are shown for clarity) in 2.5 mL Tris-HCl buffer (pH 7.2) at room temperature, ex 325 nm.

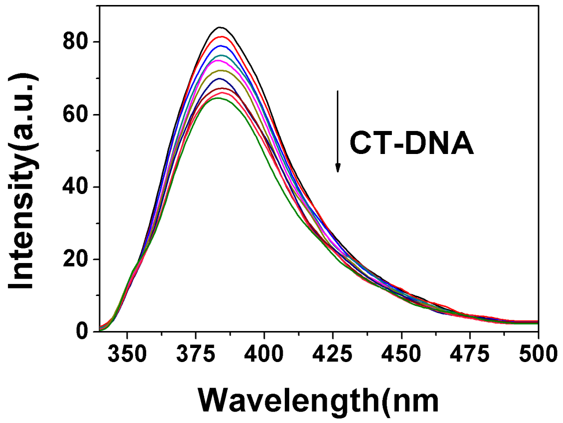


**Figure S5.** Spectrofluorimetric titration of **4d** ($2.0 \times 10^{-3}$ M) with CT DNA of increasing volume (0–450 μL) in 2.5 mL Tris-HCl buffer (pH 7.2) at room temperature, ex 325 nm.

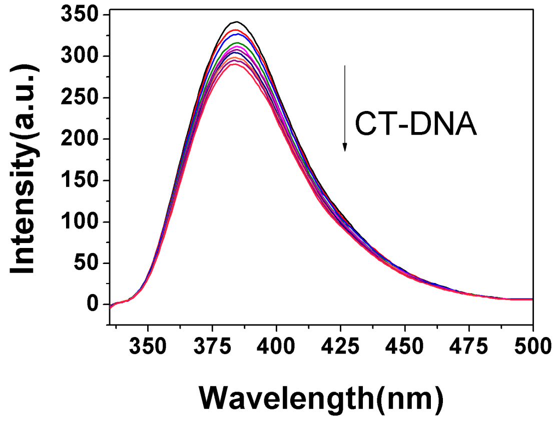


**Figure S6.** Spectrofluorimetric titration of **8e** ($2.0 \times 10^{-3}$ M) with CT DNA of increasing volume (0–450 μL) in 2.5 mL Tris-HCl buffer (pH 7.2) at room temperature, ex 325 nm.

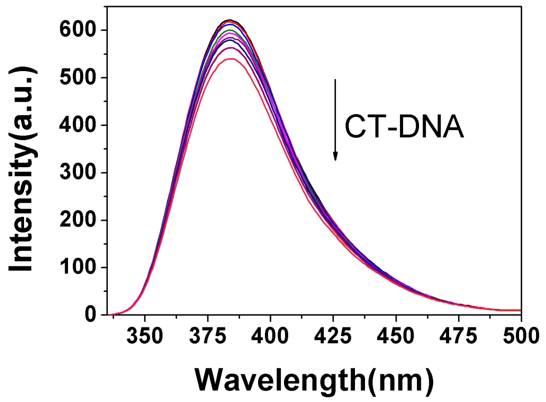


**Figure S7.** Spectrofluorimetric titration of **8f** ($2.0 \times 10^{-3}$ M) with CT DNA of increasing volume (0–450 μL) in 2.5 mL Tris-HCl buffer (pH 7.2) at room temperature, ex 325 nm.

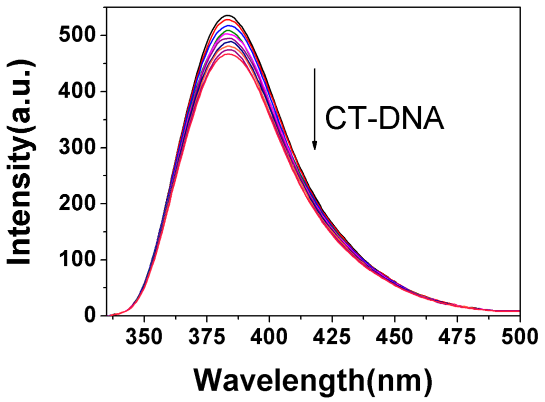


**Figure S8.** Spectrofluorimetric titration of **8g** ($2.0 \times 10^{-3}$ M) with CT DNA of increasing volume (0–450 μL) in 2.5 mL Tris-HCl buffer (pH 7.2) at room temperature, ex 325 nm.

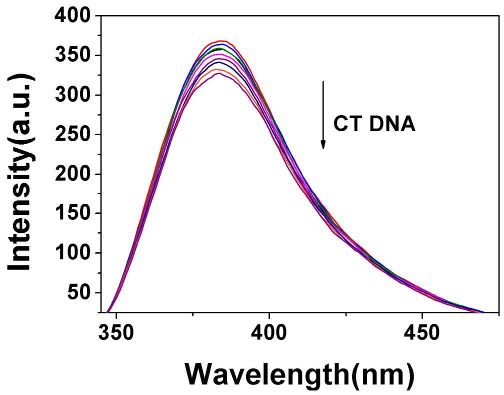


**Figure S9.** Spectrofluorimetric titration of **8h** ($2.0 \times 10^{-3}$ M) with CT DNA of increasing volume (0–450 μL, only 0, 50, 150, 200, 250, 350, 400 and 450 μL are shown for clarity) in 2.5 mL Tris-HCl buffer (pH 7.2) at roomtemperature, ex 325 nm.

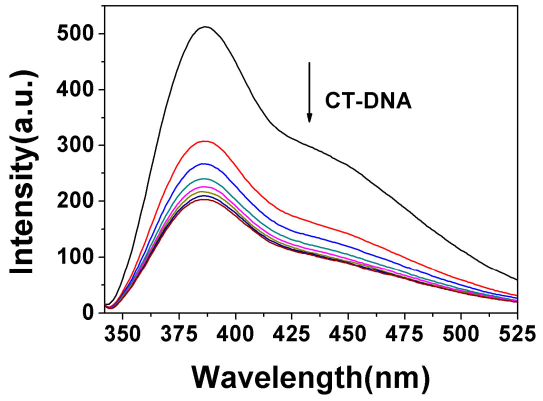


**Figure S10.** Spectrofluorimetric titration of **8i** ($2.0 \times 10^{-3}$ M) with CT DNA of increasing volume (0, 50, 60, 70, 80, 90, 100, 110, 120, 130 μL) in 2.5 mL Tris-HCl buffer (pH 7.2) at room temperature, ex 325 nm.

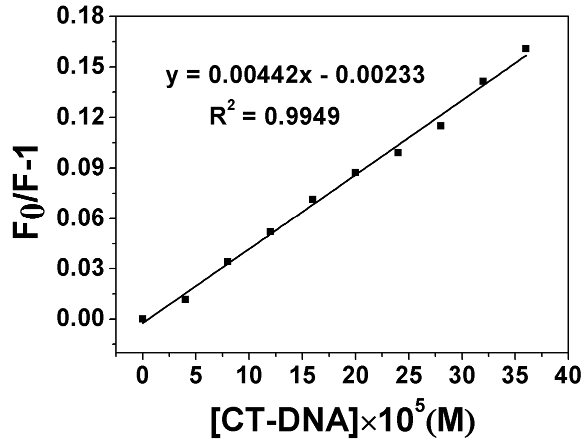


**Figure S11.** Plot of [DNA] × $10^5$ *vs.* ($F_0/F$-1) of compound **2**.

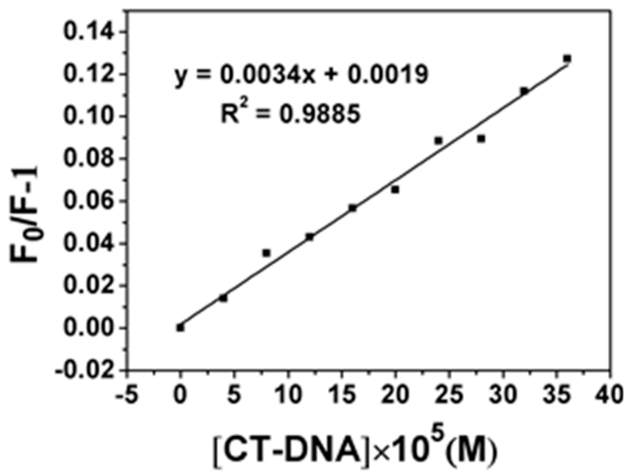


**Figure S12.** Plot of [DNA] × $10^5$ *vs.* ($F_0/F$-1) of compound **8a**.

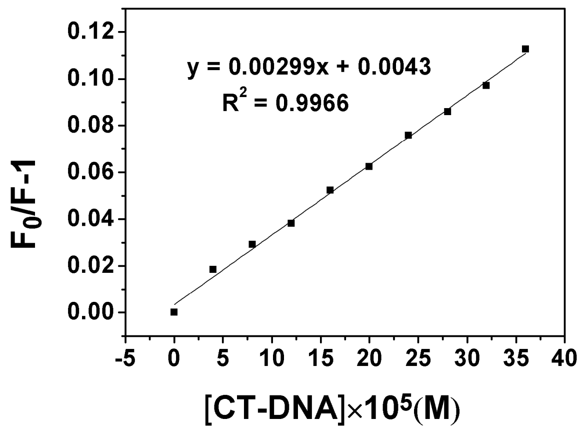


**Figure S13.** Plot of [DNA] × $10^5$ *vs.* ($F_0/F$-1) of compound **8b**.

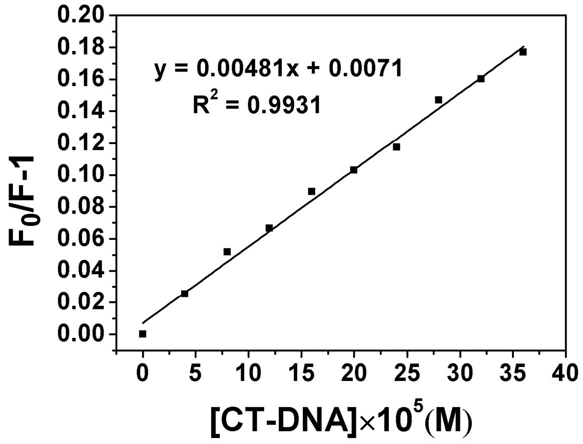


**Figure S14.** Plot of [DNA] × $10^5$ *vs.* ($F_0/F$-1) of compound **8c**.

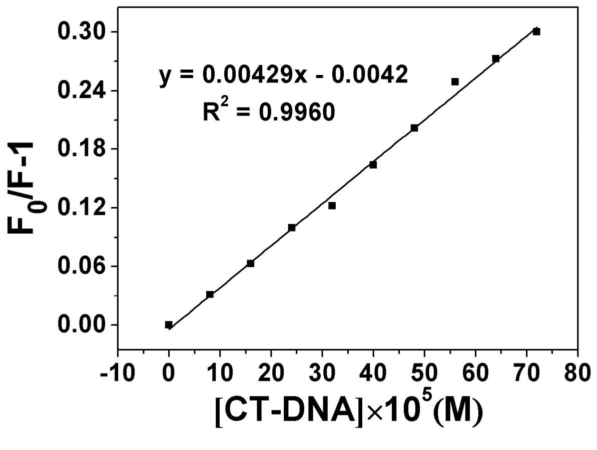


**Figure S15.** Plot of [DNA] × $10^5$ *vs.* ($F_0/F$-1) of compound **8d**.

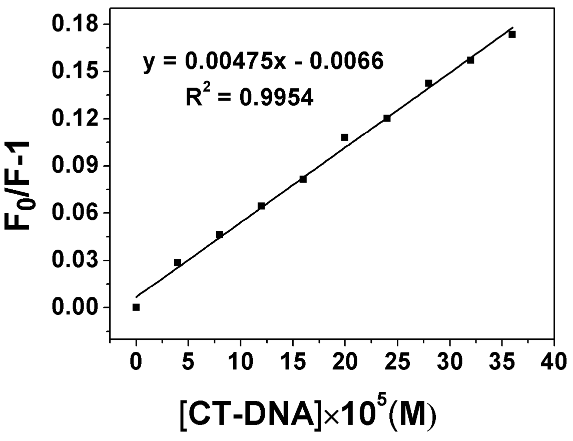


**Figure S16.** Plot of [DNA] × $10^5$ *vs.* ($F_0/F$-1) of compound **8e**.

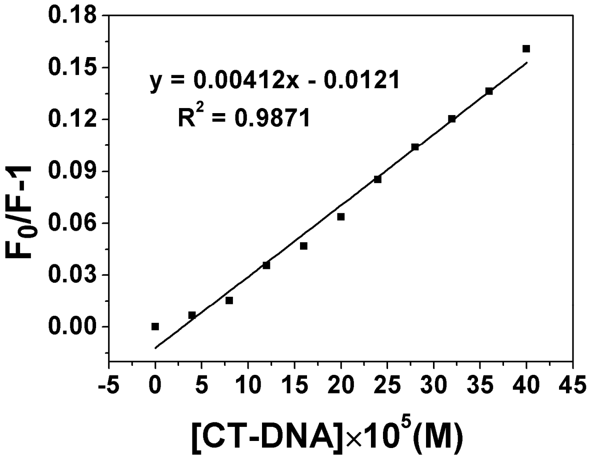


**Figure S17.** Plot of [DNA] × $10^5$ *vs.* ($F_0/F$-1) of compound **8f**.

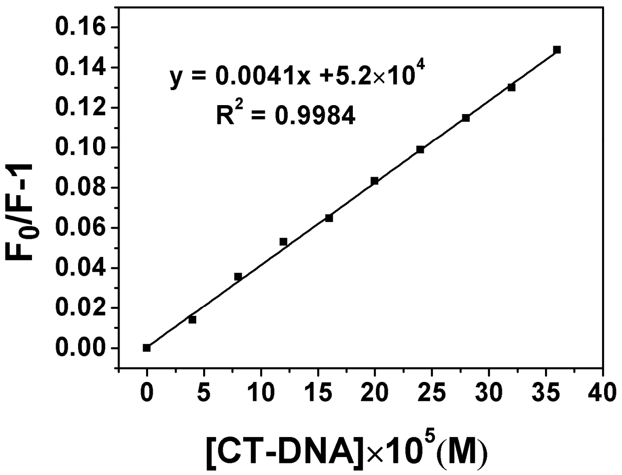


**Figure S18.** Plot of [DNA] × $10^5$ *vs.* ($F_0/F$-1) of compound **8g**.

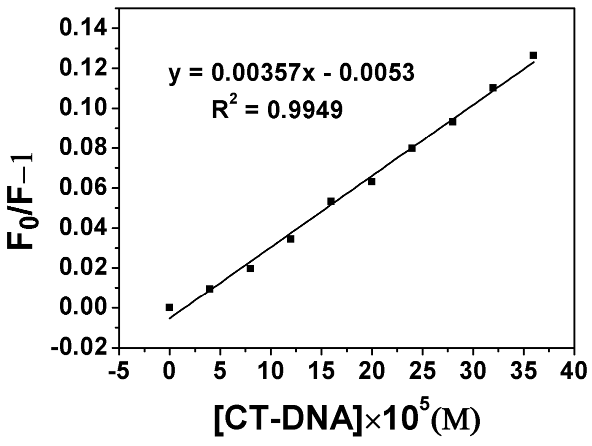


**Figure S19.** Plot of [DNA] × $10^5$ *vs*. ($F_0/F$-1) of compound **8h**.

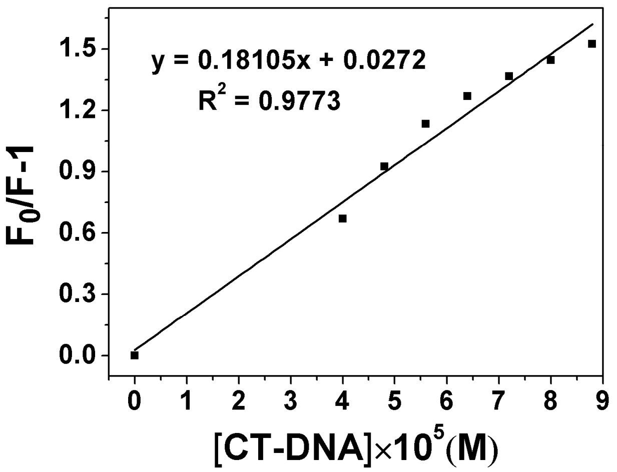


**Figure S20.** Plot of [DNA] × $10^5$ *vs*. ($F_0/F$-1) of compound **8i**.

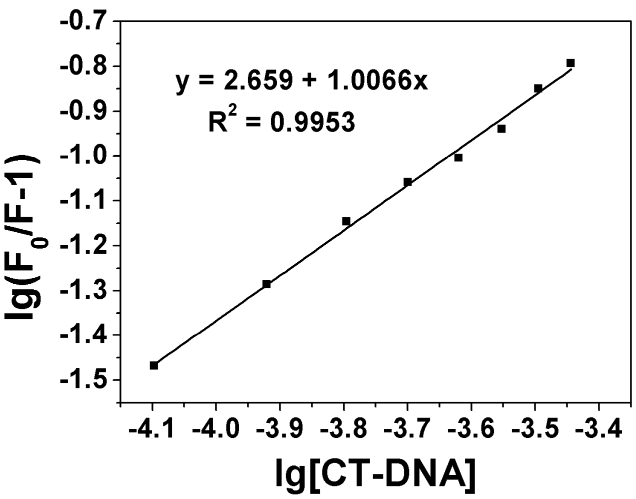


**Figure S21.** Plot of lg[DNA] *vs*. lg($F_0/F$-1), $K_b = 4.56 \times 10^2$ $M^{-1}$ of compound **2**.

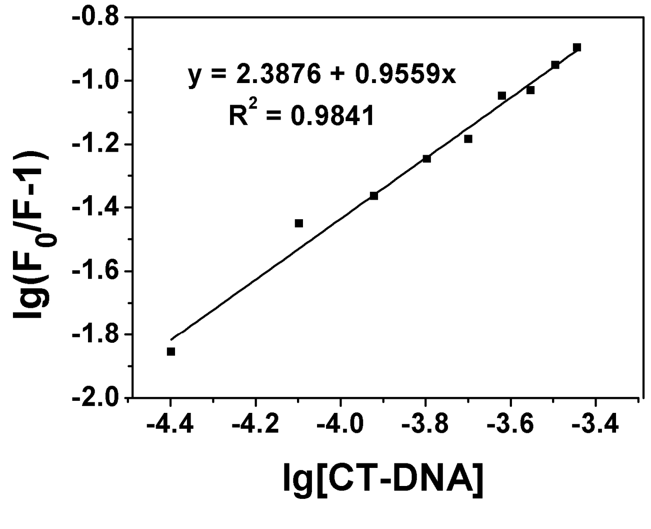


**Figure S22.** Plot of lg[DNA] *vs.* lg($F_0$/$F$-1), $K_b$ = 2.44 × $10^2$ $M^{-1}$ of compound **8a**.

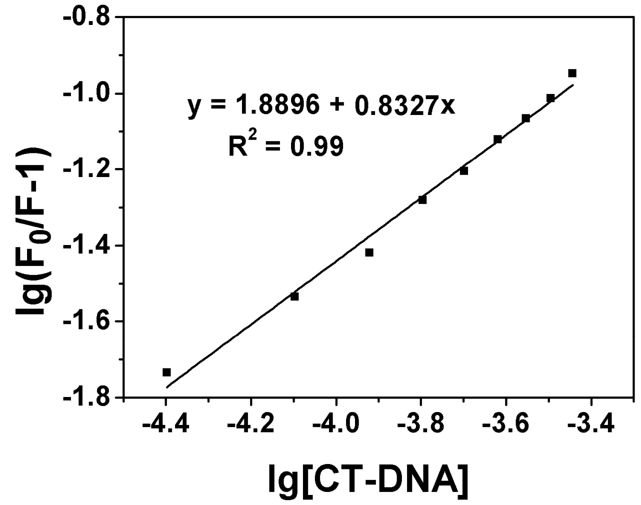


**Figure S23.** Plot of lg[DNA] *vs.* lg($F_0$/$F$-1), $K_b$ = 77.5 $M^{-1}$ of compound **8b**.

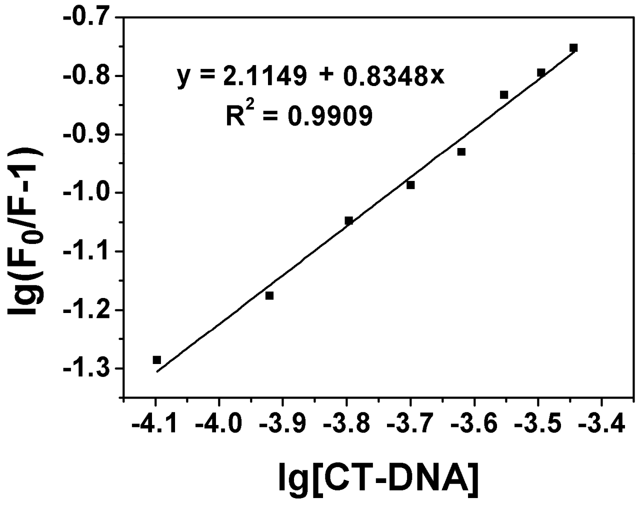


**Figure S24.** Plot of lg[DNA] *vs.* lg($F_0$/$F$-1), $K_b$ = 1.30 × $10^2$ $M^{-1}$ of compound **8c**.

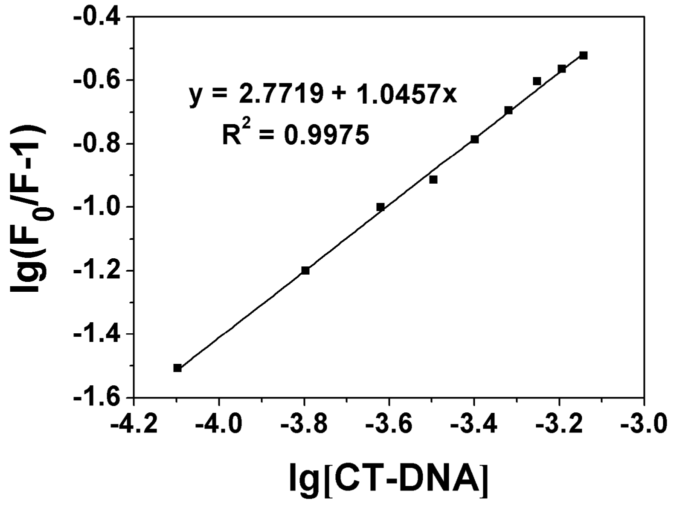


**Figure S25.** Plot of lg[DNA] *vs.* lg($F_0/F$-1), $K_b = 5.91 \times 10^2$ $M^{-1}$ of compound **8d**.

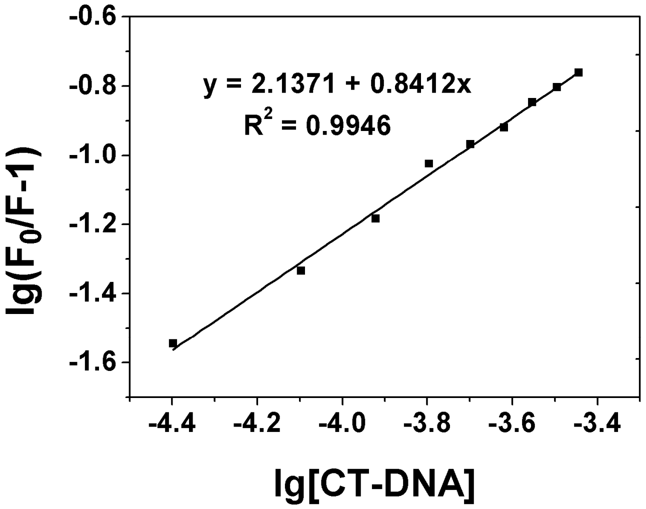


**Figure S26.** Plot of lg[DNA] *vs.* lg($F_0/F$-1), $K_b = 1.37 \times 10^2$ $M^{-1}$ of compound **8e**.

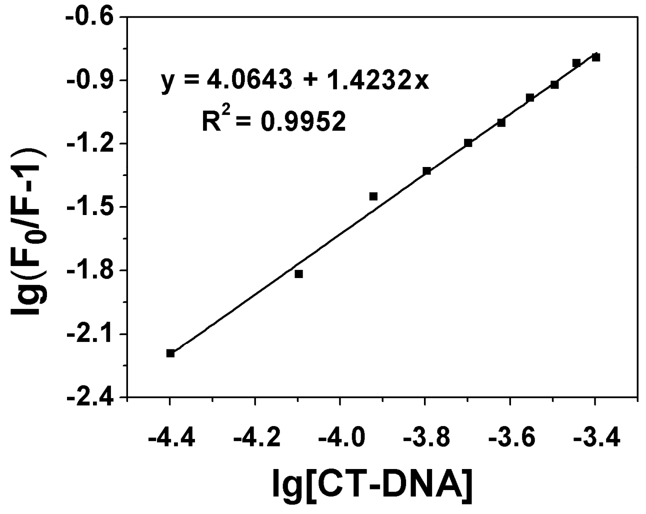


**Figure S27.** Plot of lg[DNA] *vs.* lg($F_0/F$-1), $K_b = 1.16 \times 10^4$ $M^{-1}$ of compound **8f**.

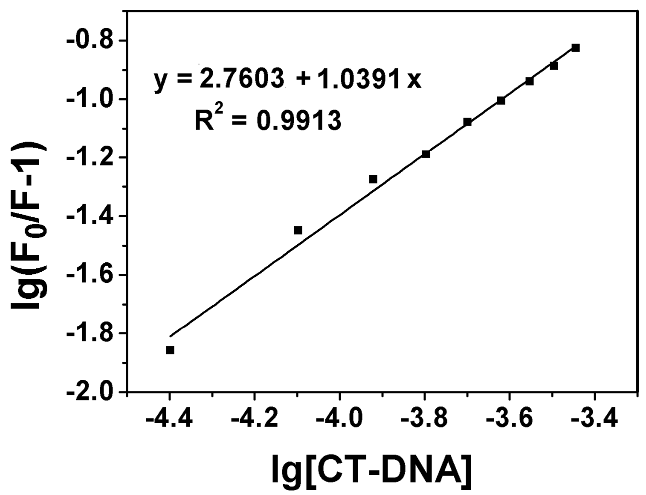


**Figure S28.** Plot of lg[DNA] *vs.* lg($F_0/F$-1), $K_b = 5.76 \times 10^2$ $M^{-1}$ of compound **8g**.

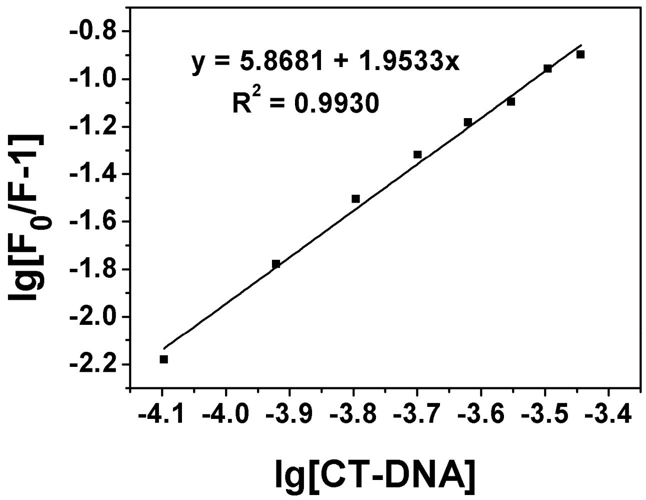


**Figure S29.** Plot of lg[DNA] *vs.* lg($F_0/F$-1), $K_b = 7.38 \times 10^5$ $M^{-1}$ of compound **8h**.

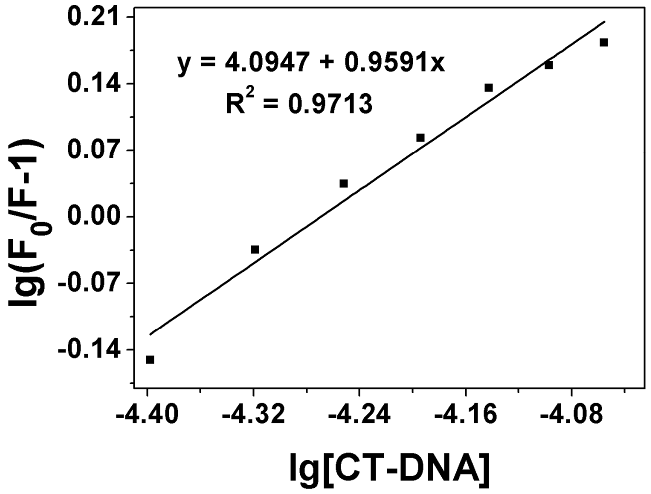


**Figure S30.** Plot of lg[DNA] *vs.* lg($F_0/F$-1), $K_b = 1.24 \times 10^4$ $M^{-1}$ of compound **8i**.

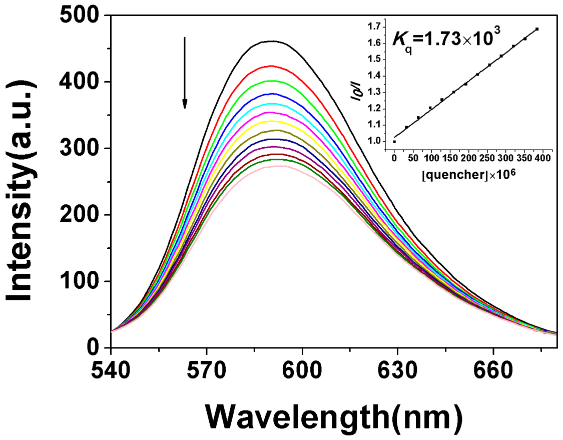


**Figure S31.** Emission spectra of DNA-GelRed (165 μM), in the presence of 0, 40, 80, 120, 160, 200, 240, 280, 320, 360 and 400 μM of compound **8f**. Arrow indicates the changes in the emission intensity as a function of compound concentration. Inset: SterneVolmer plot of the fluorescence titration data corresponding to the compound **8f**.

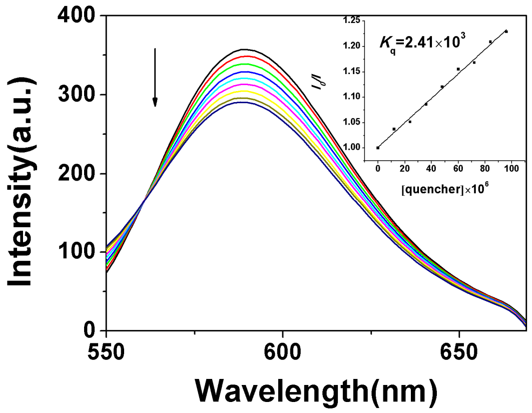


**Figure S32.** Emission spectra of DNA-GelRed (165 μM), in the presence of 0, 15, 30, 45, 60, 75, 90, 105 and 120 μM of compound **8h**. Arrow indicates the changes in the emission intensity as a function of compound concentration. Inset: SterneVolmer plot of the fluorescence titration data corresponding to the compound **8h**.

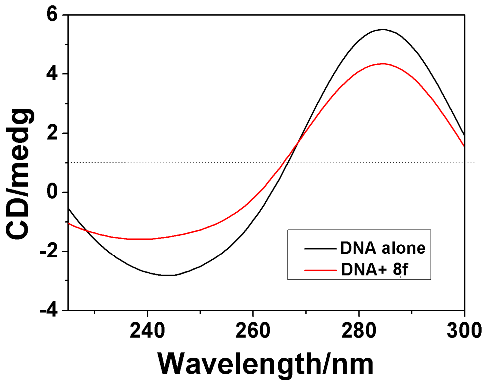


**Figure S33.** CD spectra of CT-DNA (3 mL solution, 1.5 × $10^{-4}$ M) in the absence and presence of compound **8f** (1.5 × $10^{-5}$ M).

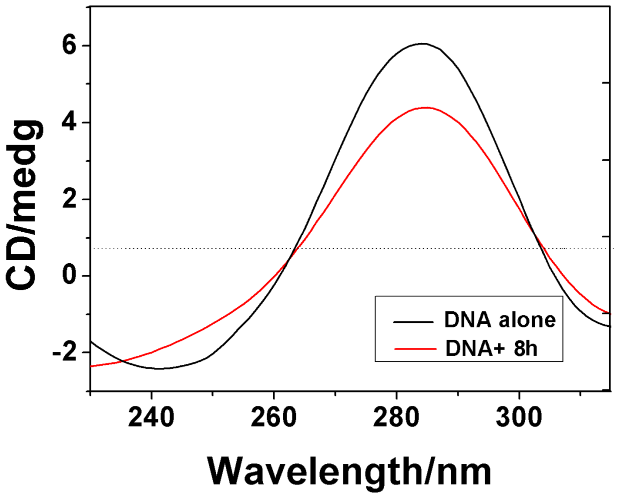


**Figure S34.** CD spectra of CT-DNA (3 mL solution, 1.5 × $10^{-4}$ M) in the absence and presence of compound **8h** (1.5 × $10^{-5}$ M).

$^1$H-NMR (500 MHz, $CDCl_3$) for compound **3**: δ 7.42 (d, *J* = 8.8 Hz, 1H), 6.75 (dd, *J* = 8.8, 2.5 Hz, 1H), 6.70 (d, *J* = 2.5 Hz, 1H), 6.09 (d, *J* = 1.1 Hz, 1H), 4.27 (t, *J* = 6.3 Hz, 2H), 2.76 (t, *J* = 6.3 Hz, 2H), 2.34 (d, *J* = 1.1 Hz, 3H).

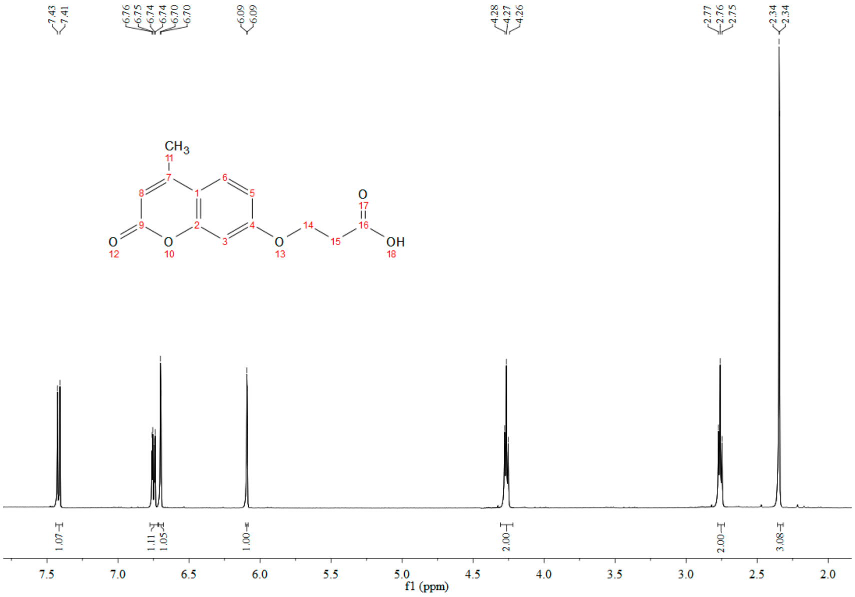


**Figure S35.** Spectrum of compound **3**.

$^{1}$H-NMR (500 MHz, $CDCl_3$) for compound **8a**: δ 7.53–7.49 (m, 2H), 7.42 (d, *J* = 8.8 Hz, 1H), 7.29 (qd, *J* = 7.9, 1.3 Hz, 3H), 6.75 (dd, *J* = 8.8, 2.5 Hz, 1H), 6.70 (d, *J* = 2.5 Hz, 1H), 6.09 (d, *J* = 1.1 Hz, 1H), 5.61 (dd, *J* = 21.0, 9.7 Hz, 1H), 4.30–4.23 (m, 2H), 4.18–3.59 (m, 4H), 2.76 (t, *J* = 6.3 Hz, 2H), 2.34 (d, *J* = 1.1 Hz, 3H), 1.31 (t, *J* = 7.1 Hz, 3H), 1.05 (t, *J* = 7.1 Hz, 3H). $^{31}$P-NMR (202 MHz, $CDCl_3$) δ (ppm) 21.49 (s). HRMS for $C_{24}H_{29}NO_7P$ ($[M + H]^+$): calcd 474.16816: found 474.16656.

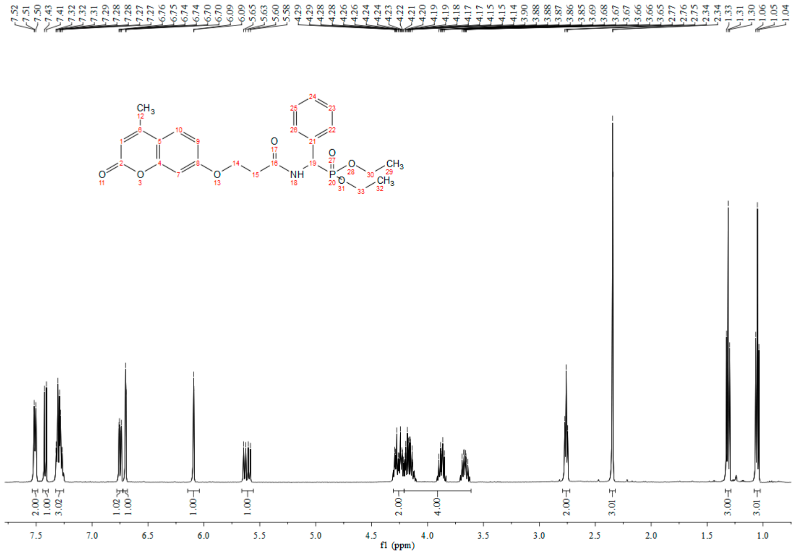


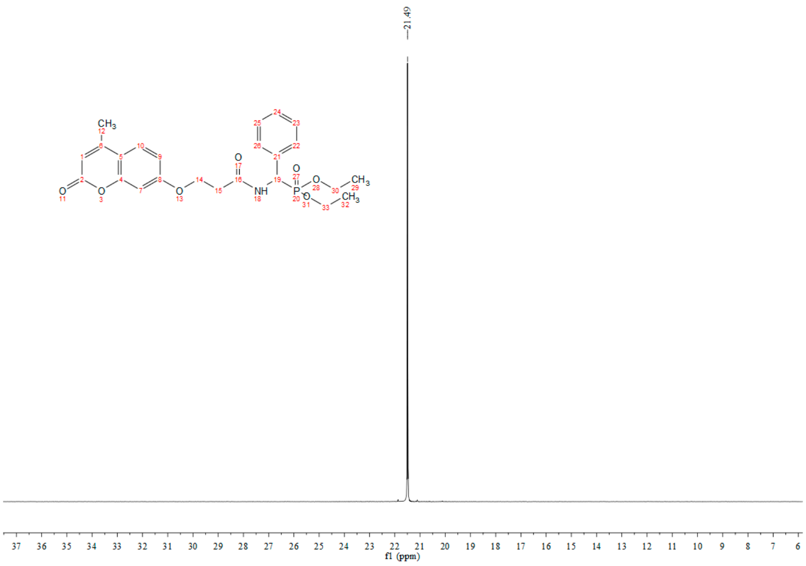


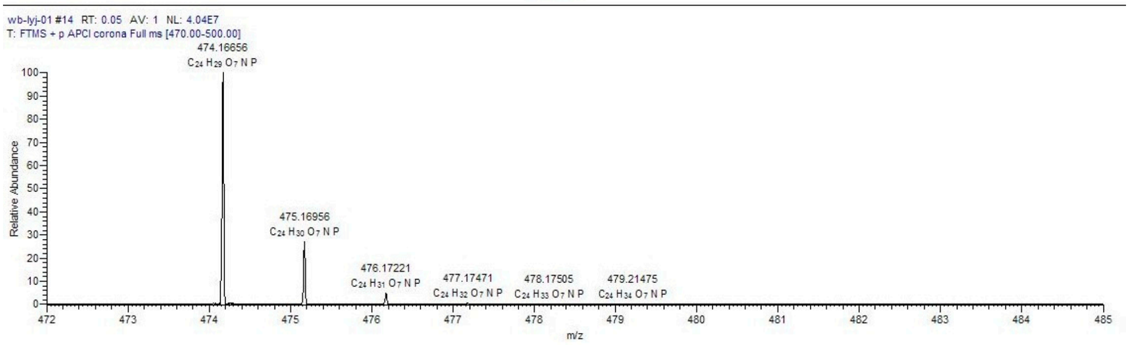


**Figure S36.** Spectrum of compound **8a**.

$^{1}$H-NMR (500 MHz, $CDCl_3$) for compound **8b**: δ (ppm) 8.30 (d, *J* = 7.4 Hz, 1H), 7.46 (ddd, *J* = 8.6, 5.1, 2.0 Hz, 2H), 7.41 (d, *J* = 8.8 Hz, 1H), 6.97 (t, *J* = 8.6 Hz, 2H), 6.73 (dd, *J* = 8.8, 2.5 Hz, 1H), 6.70 (d, *J* = 2.4 Hz, 1H), 6.08 (d, *J* = 1.1 Hz, 1H), 5.56 (dd, *J* = 21.0, 9.6 Hz, 1H), 4.25 (ddd, *J* = 15.5, 9.3, 3.1 Hz, 2H), 4.18–3.65 (m, 4H), 2.74 (t, *J* = 6.2 Hz, 2H), 2.34 (d, *J* = 1.1 Hz, 3H), 1.29 (t, *J* = 7.1 Hz, 3H), 1.07 (t, *J* = 7.1 Hz, 3H).$^{31}$P-NMR (202 MHz, $CDCl_3$) δ (ppm) 21.20 (d, *J* = 4.3 Hz). HRMS for $C_{24}H_{28}NO_7FP$ ($[M + H]^+$): calcd 492.15874: found 492.15732.

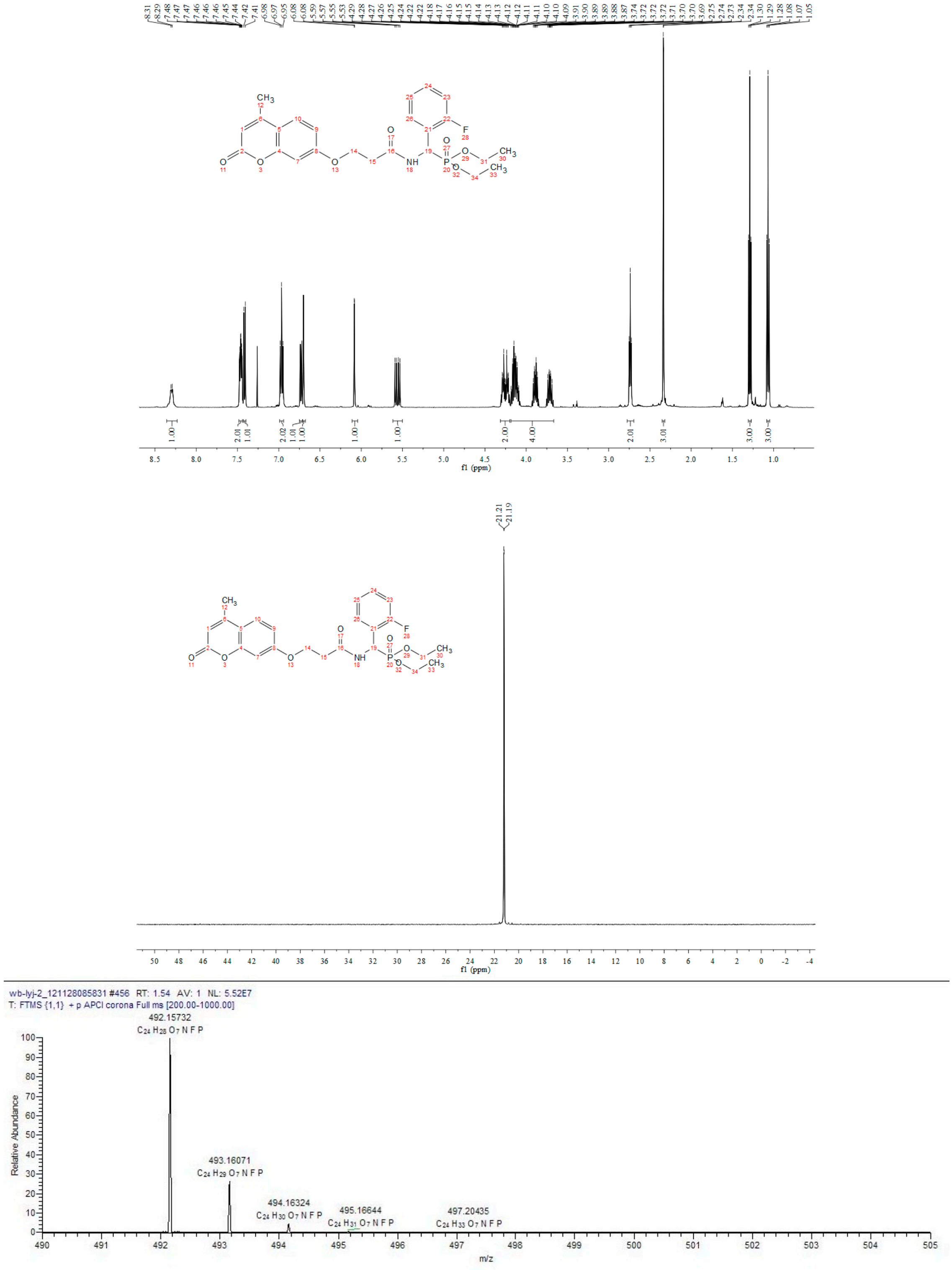


**Figure S37.** Spectrum of compound **8b**.

$^{1}$H-NMR (500 MHz, $CDCl_3$) for compound **8c**: δ (ppm) 8.01 (s, 1H), 7.47 (d, $J$ = 8.8 Hz, 1H), 7.42–7.21 (m, 4H), 6.81 (dd, $J$ = 8.8, 2.5 Hz, 1H), 6.75 (d, $J$ = 2.4 Hz, 1H), 6.22–6.16 (m, 1H), 6.14 (s, 1H), 4.36–4.30 (m, 2H), 4.30–3.64 (m, 4H), 2.79 (td, $J$ = 6.1, 2.6 Hz, 2H), 2.39 (d, $J$ = 0.8 Hz, 3H), 1.36 (t, $J$ = 7.1 Hz, 3H), 1.06 (t, $J$ = 7.0 Hz, 3H). $^{31}$P-NMR (202 MHz, $CDCl_3$) δ (ppm) 20.72(s). HRMS for $C_{24}H_{28}NO_7PCl$ ($[M + H]^+$): calcd 508.12919; found 508.12781.

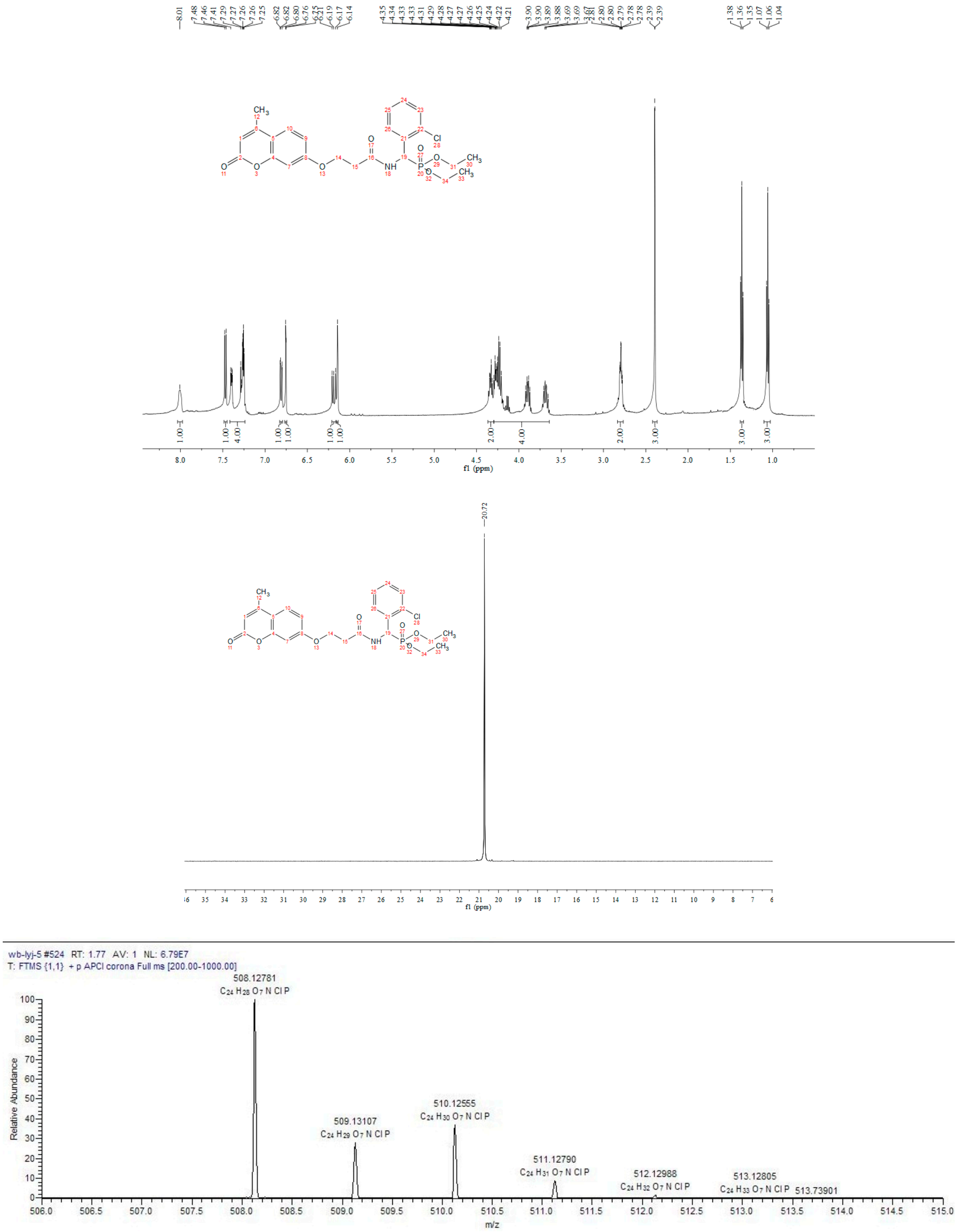


**Figure S38.** Spectrum of compound **8c**.

$^{1}$H-NMR (500 MHz, $CDCl_3$) for compound **8d**: δ (ppm) 8.21 (s, 1H), 7.46 (dd, *J* = 8.5, 2.2 Hz, 1H), 7.42 (d, *J* = 8.3 Hz, 2H), 7.29 (dd, *J* = 8.3, 1.6 Hz, 2H), 6.78 (d, *J* = 2.5 Hz, 1H), 6.76 (s, 1H), 6.13 (d, *J* = 1.0 Hz, 1H), 5.58 (dd, *J* = 21.3, 9.5 Hz, 1H), 4.40–4.23 (m, 2H), 4.21–3.72 (m, 4H), 2.78 (t, *J* = 6.0 Hz, 2H), 2.38 (d, *J* = 1.0 Hz, 3H), 1.32 (t, *J* = 7.0 Hz, 3H), 1.12 (dd, *J* = 7.7, 6.4 Hz, 3H). $^{31}$P-NMR (202 MHz, $CDCl_3$) δ (ppm) 21.21 (s). HRMS for $C_{24}H_{28}NO_7$ PCl ($[M + H]^+$): calcd 508.12919; found 508.12775.

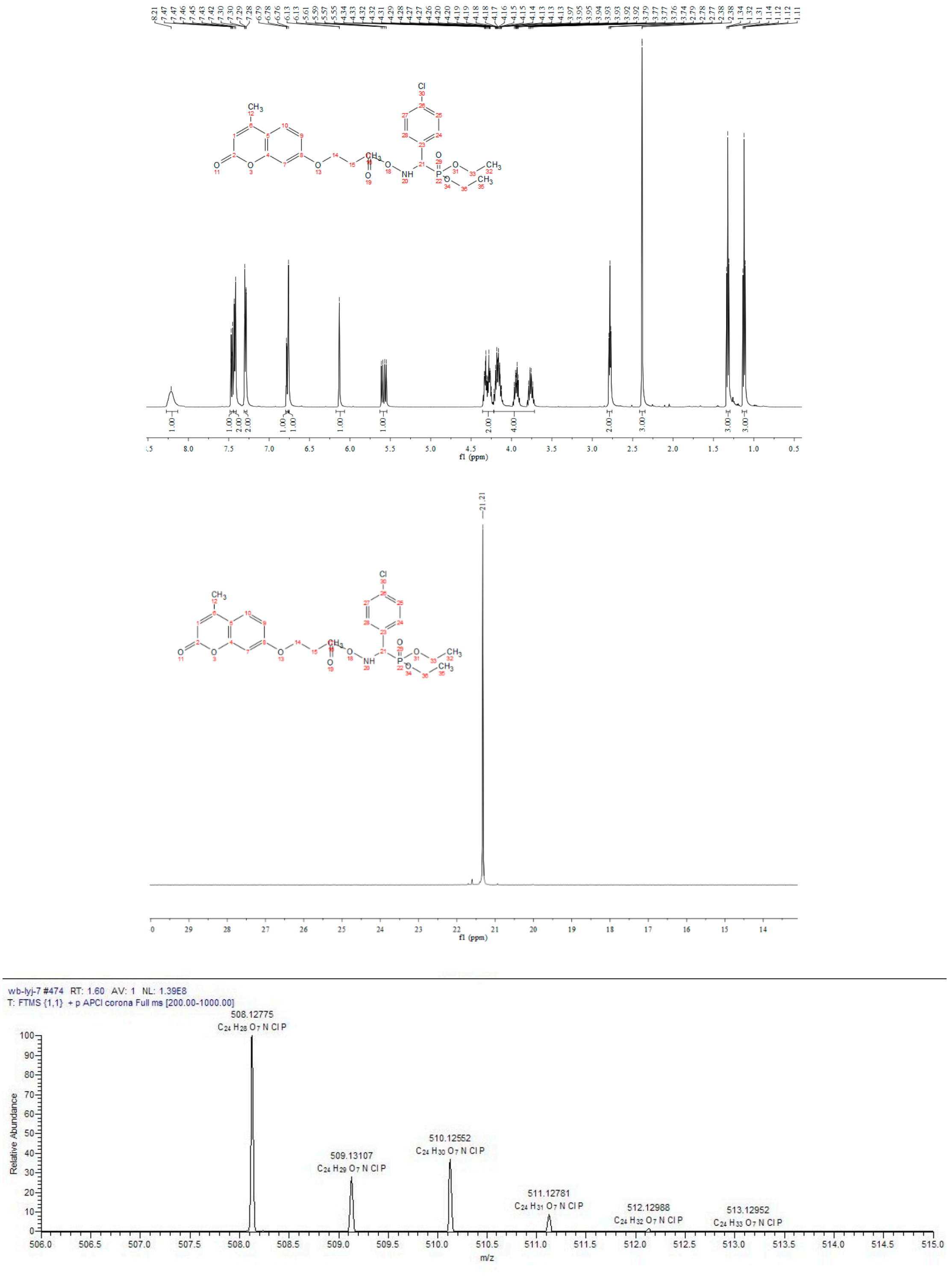


**Figure S39.** Spectrum of compound **8d**.

$^{1}$H-NMR (500 MHz, $CDCl_3$) for compound **8e**: δ (ppm)8.67 (s, 1H), 7.75 (d, *J* = 7.8 Hz, 1H), 7.53 (d, *J* = 8.0 Hz, 1H), 7.40 (dd, *J* = 8.8, 1.2 Hz, 1H), 7.27 (dd, *J* = 12.7, 5.2 Hz, 1H), 7.12 (dd, *J* = 11.0, 4.3 Hz, 1H), 6.73 (dd, *J* = 8.8, 2.4 Hz, 1H), 6.67 (d, *J* = 2.3 Hz, 1H), 6.20 (dd, *J* = 21.0, 9.3 Hz, 1H), 6.07 (s, 1H), 4.34–4.23 (m, 2H), 4.24–3.58 (m, 4H), 2.77 (q, *J* = 6.1 Hz, 2H), 2.33 (s, 3H), 1.34 (t, *J* = 7.1 Hz, 3H), 1.02 (t, *J* = 7.1 Hz, 3H).$^{31}$P-NMR (202 MHz, $CDCl_3$) δ (ppm) 20.85 (s). HRMS for $C_{24}H_{28}NO_7PBr$ ($[M + H]^+$): calcd 552.07868; found 552.07666.

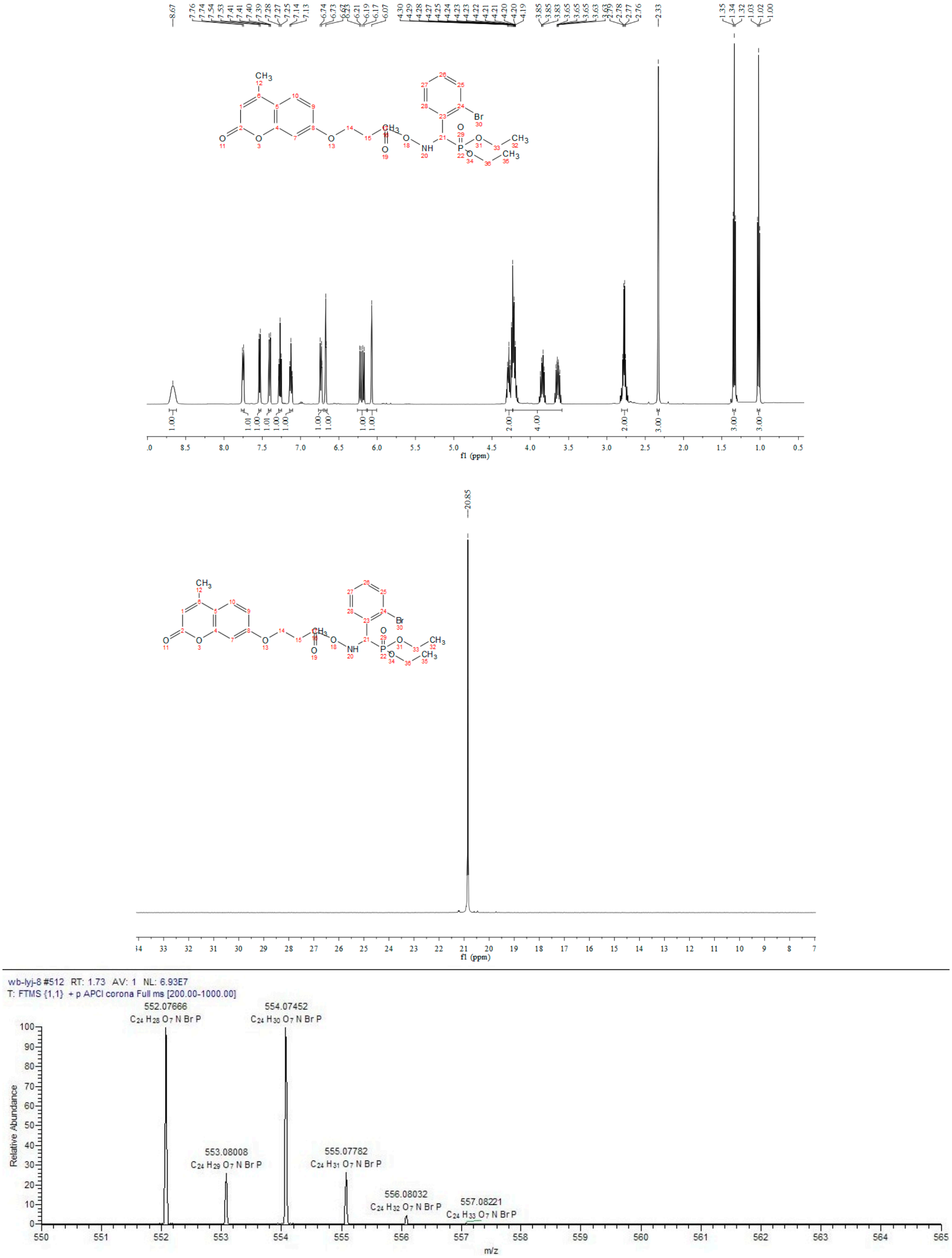


**Figure S40.** Spectrum of compound **8e**.

$^{1}$H-NMR (500 MHz, $CDCl_3$) for compound **8f**: δ (ppm) 8.36 (d, $J$ = 5.7 Hz, 1H), 7.67 (d, $J$ = 1.5 Hz, 1H), 7.44 (dd, $J$ = 15.1, 8.4 Hz, 2H), 7.20 (t, $J$ = 7.8 Hz, 1H), 6.79 (dd, $J$ = 8.8, 2.4 Hz, 1H), 6.74 (d, $J$ = 2.4 Hz, 1H), 6.13 (d, $J$ = 0.7 Hz, 1H), 5.58 (dd, $J$ = 21.3, 9.5 Hz, 1H), 4.30 (dq, $J$ = 9.3, 6.4 Hz, 2H), 4.26–3.73 (m, 4H), 2.79 (t, $J$ = 6.1 Hz, 2H), 2.38 (d, $J$ = 0.5 Hz, 3H), 1.33 (t, $J$ = 7.1 Hz, 3H), 1.12 (t, $J$ = 7.1 Hz, 3H). $^{31}$P-NMR (202 MHz, $CDCl_3$) δ (ppm) 21.71 (s). HRMS for $C_{24}H_{28}NO_7PBr$ ($[M + H]^+$): calcd 552.07868; found 552.07642.

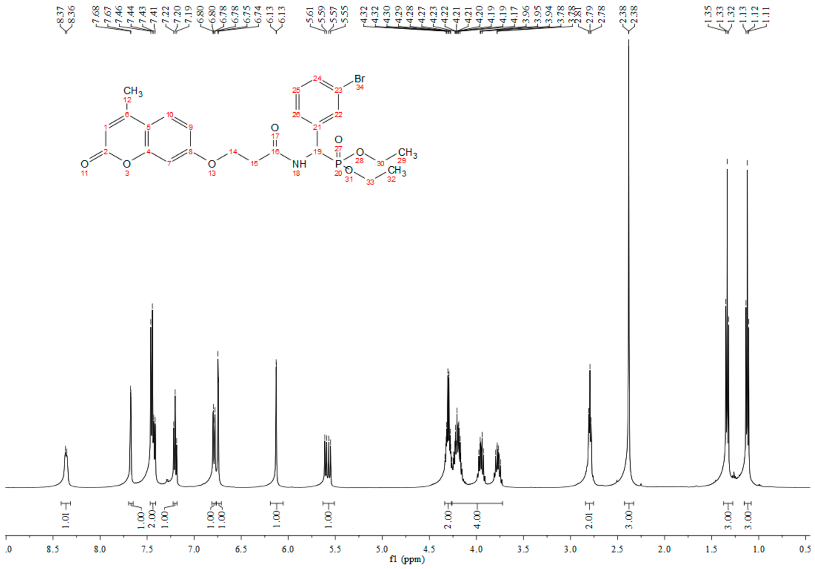


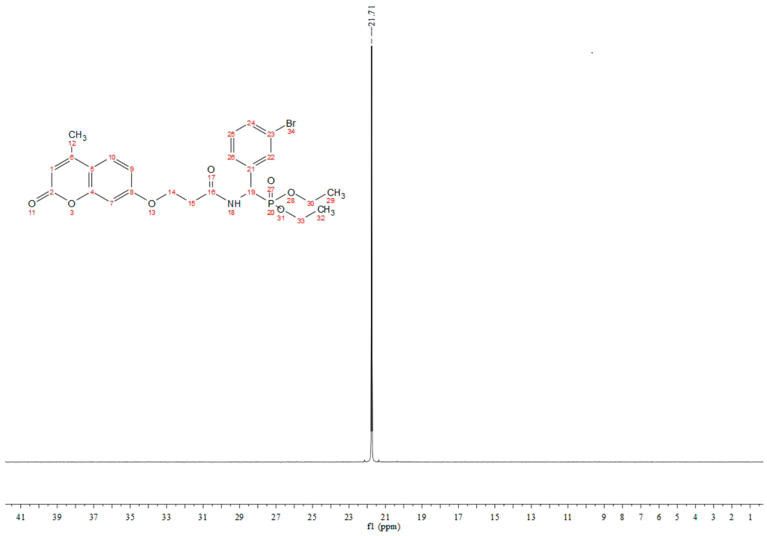


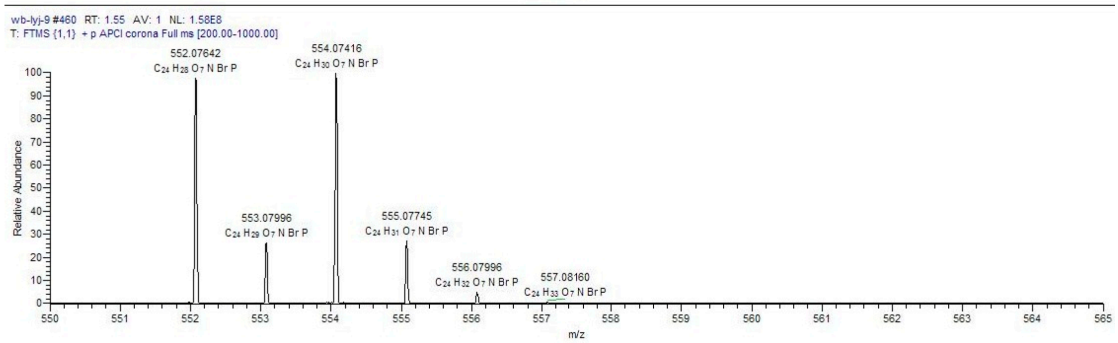


**Figure S41.** Spectrum of compound **8f**.

$^{1}$H-NMR (500 MHz, $CDCl_3$) for compound **8g**: δ (ppm) 8.23 (dd, *J* = 9.5, 4.1 Hz, 1H), 7.43 (d, *J* = 8.1 Hz, 2H), 7.41 (s, 1H), 7.33 (dd, *J* = 8.5, 2.0 Hz, 2H), 6.75 dd, *J* = 8.8, 2.4 Hz, 1H), 6.73 (d, *J* = 2.3 Hz, 1H), 6.10 (d, *J* = 1.2 Hz, 1H), 5.54 (dd, *J* = 21.3, 9.5 Hz, 1H), 4.32–4.21 (m, 2H), 4.19–3.70 (m, 4H), 2.75 (t, *J* = 6.1 Hz, 2H), 2.35 (d, *J* = 1.2 Hz, 3H), 1.29 (t, *J* = 7.1 Hz, 3H), 1.09 (t, *J* = 7.1 Hz, 3H). $^{31}$P-NMR (202 MHz, $CDCl_3$) δ (ppm) 20.87 (s). HRMS for $C_{24}H_{28}NO_7PBr$ ($[M + H]^+$): calcd 552.07868; found 552.07629.

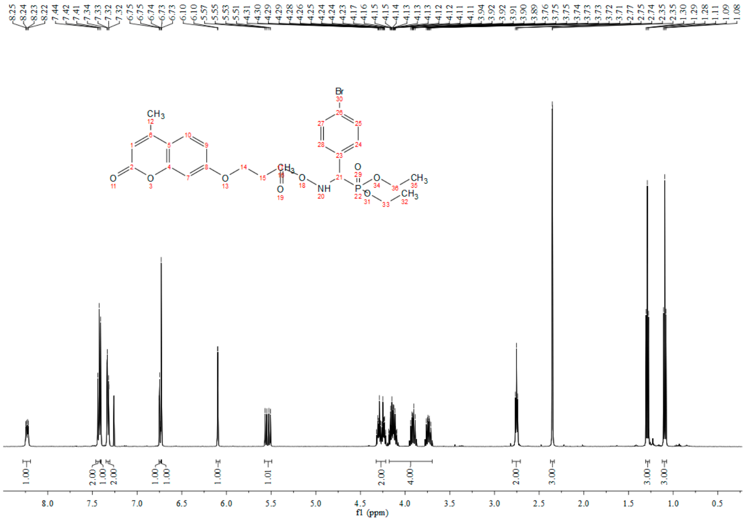


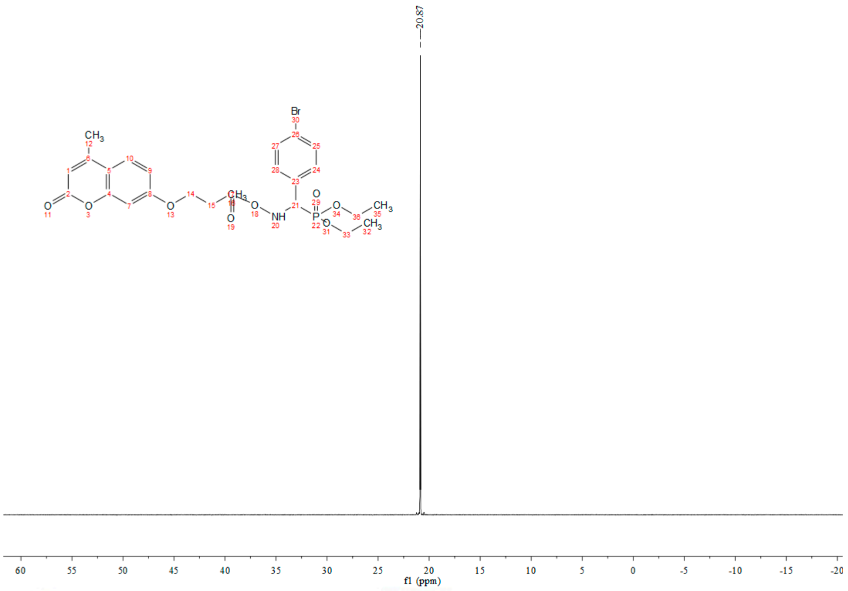


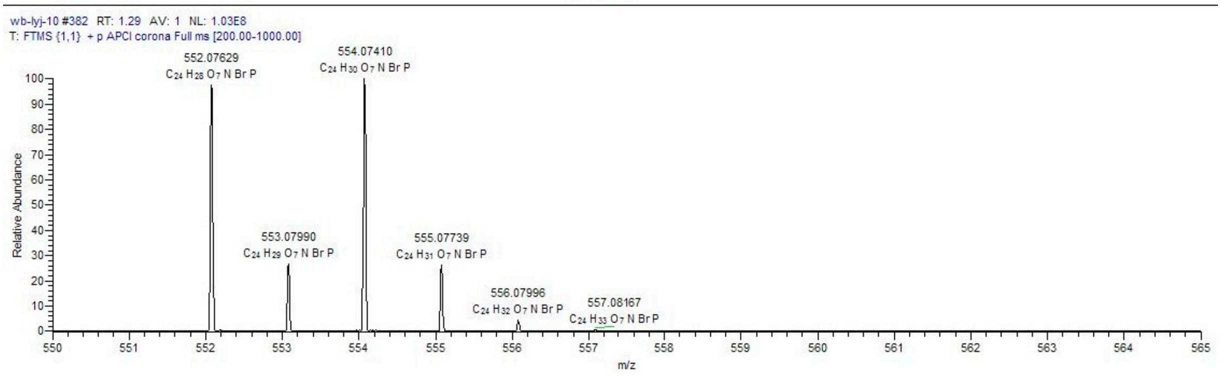


**Figure S42.** Spectrum of compound **8g**.

$^{1}$H-NMR (500 MHz, $CDCl_3$) for complex **8h**: δ (ppm) 8.08 (dd, *J* = 9.7, 2.9 Hz, 1H), 7.57–7.45 (m, 1H), 7.39 (d, *J* = 8.8 Hz, 1H), 7.24–7.18 (m, 1H), 6.91–6.81 (m, 2H), 6.74 (dd, *J* = 8.8, 2.5 Hz, 1H), 6.68 (d, *J* = 2.5 Hz, 1H), 6.14–6.08 (m, 1H), 6.06 (d, *J* = 1.2 Hz, 1H), 4.30–4.20 (m, 2H), 4.19–3.70 (m, 4H),3.82 (s, 3H), 2.73 (dd, *J* = 10.4, 6.1 Hz, 2H), 2.32 (d, *J* = 1.0 Hz, 3H), 1.28 (t, *J* = 7.1 Hz, 3H), 0.98 (t, *J* = 7.1 Hz, 3H). $^{31}$P-NMR (202 MHz, $CDCl_3$) δ (ppm) 22.05 (s). HRMS for $C_{25}H_{31}NO_8$ P ($[M + H]^+$): calcd 504.17873; found 504.17688.

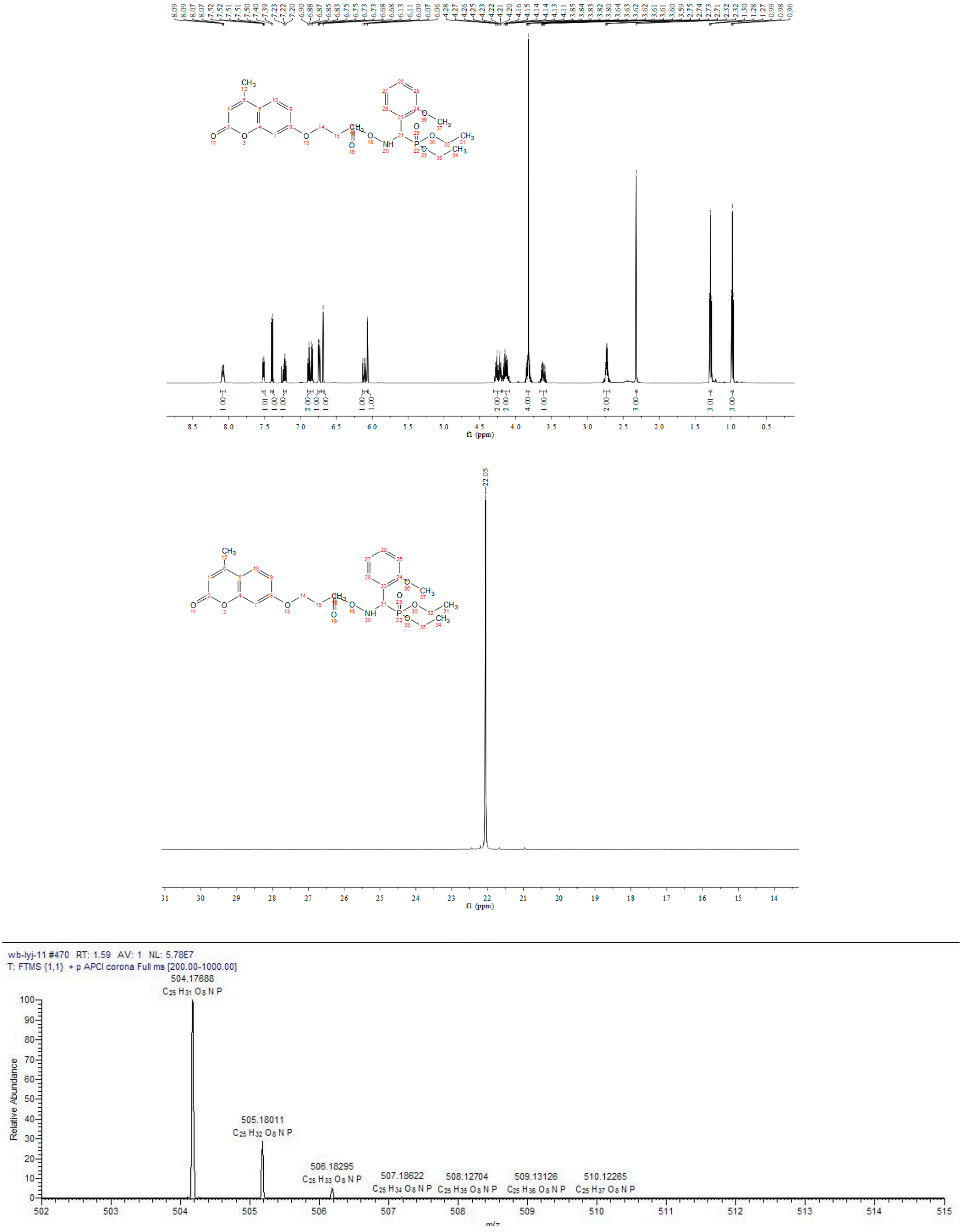


**Figure S43.** Spectrum of compound **8h**.

$^1$H-NMR (500 MHz, $CDCl_3$) for complex **8i**: δ (ppm) 8.46 (s, 1H), 7.98 (s, 1H), 7.79–7.71 (m, 3H), 7.63 (dd, $J$ = 8.5, 1.4 Hz, 1H), 7.47–7.39 (m, 2H), 7.32–7.27 (m, 1H), 6.67 (d, $J$ = 1.3 Hz, 1H), 6.65 (t, $J$ = 1.9 Hz, 1H), 6.07 (s, 1H), 5.80 (dd, $J$ = 21.0, 9.6 Hz, 1H), 4.27–3.59 (m, 6H) 2.76 (t, $J$ = 6.3 Hz, 2H), 2.29 (s, 3H), 1.34 (t, $J$ = 7.1 Hz, 3H), 1.02 (t, $J$ = 7.1 Hz, 3H). $^{31}$P-NMR (202 MHz, $CDCl_3$) δ (ppm) 21.44 (s). HRMS for $C_{28}H_{31}NO_7P$([M + H]$^+$): calcd 524.18381; found 524.18213.

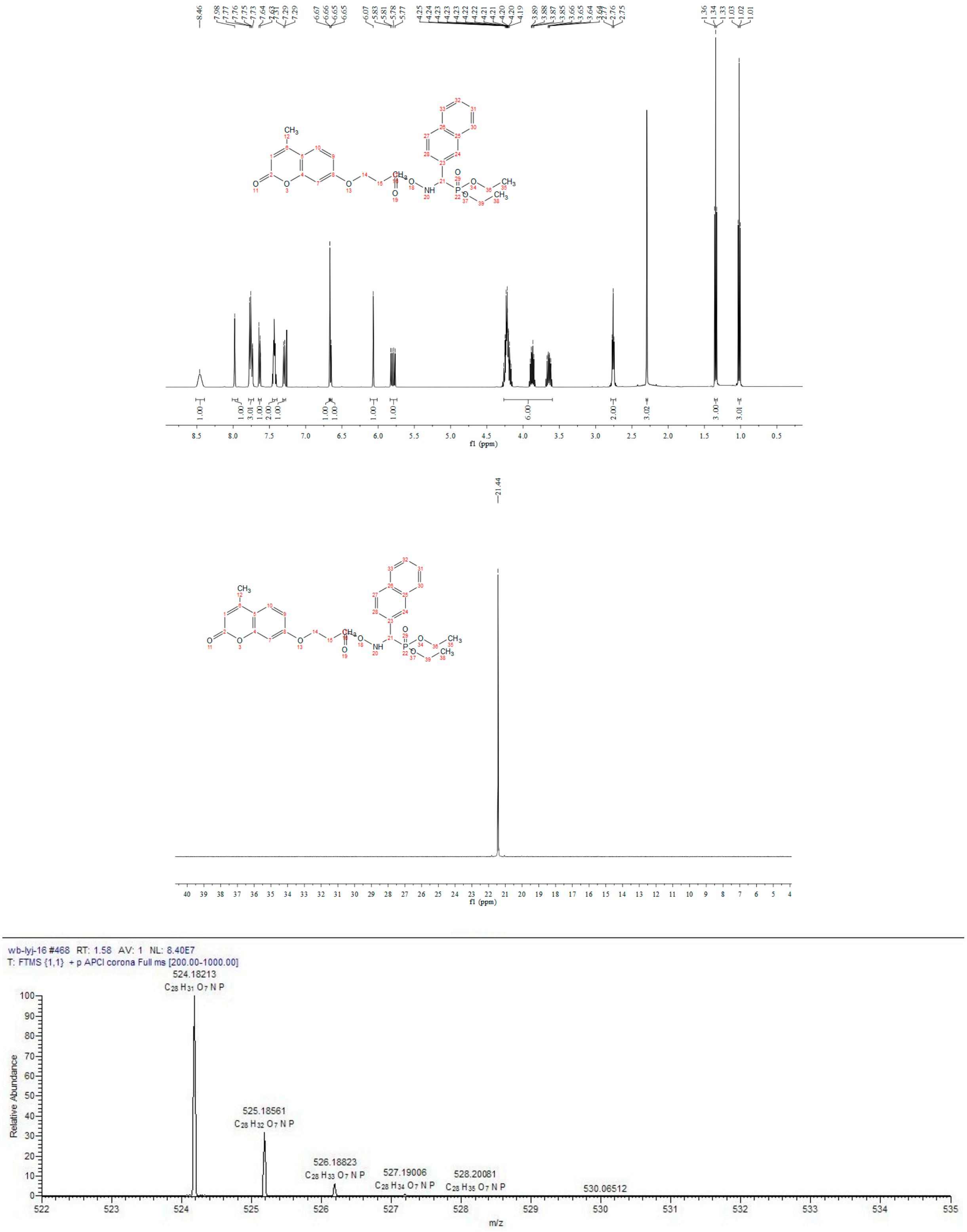


**Figure S44.** Spectrum of compound **8i**.

$^{1}$H-NMR (500 MHz, $CDCl_3$) for complex **8j**: $^{1}$H-NMR (500 MHz, $CDCl_3$) δ(ppm) 8.45 (dd, $J$ = 9.6, 3.4 Hz, 1H), 7.62–7.45 (m, 2H), 7.42 (d, $J$ = 8.8 Hz, 1H), 7.29 (dd, $J$ = 7.9, 4.1 Hz, 3H), 6.75 (dd, $J$ = 8.8, 2.5 Hz, 1H), 6.70 (d, $J$ = 2.5 Hz, 1H), 6.09 (d, $J$ = 1.1 Hz, 1H), 4.34–4.22 (m, 2H), 4.22–3.67 (m, 4H), 2.78 (td, $J$ = 6.2, 2.7 Hz, 2H), 2.34 (d, $J$ = 1.0 Hz, 3H), 2.10 (d, $J$ = 16.1 Hz, 1H), 1.31 (t, $J$ = 7.1 Hz, 3H), 1.09 (t, $J$ = 7.1 Hz, 3H). $^{31}$P-NMR (202 MHz, $CDCl_3$) δ (ppm) 21.33 (s). HRMS for $C_{25}H_{31}NO_7P$ ($[M + H]^+$): calcd 488.18381; found 488.18231.

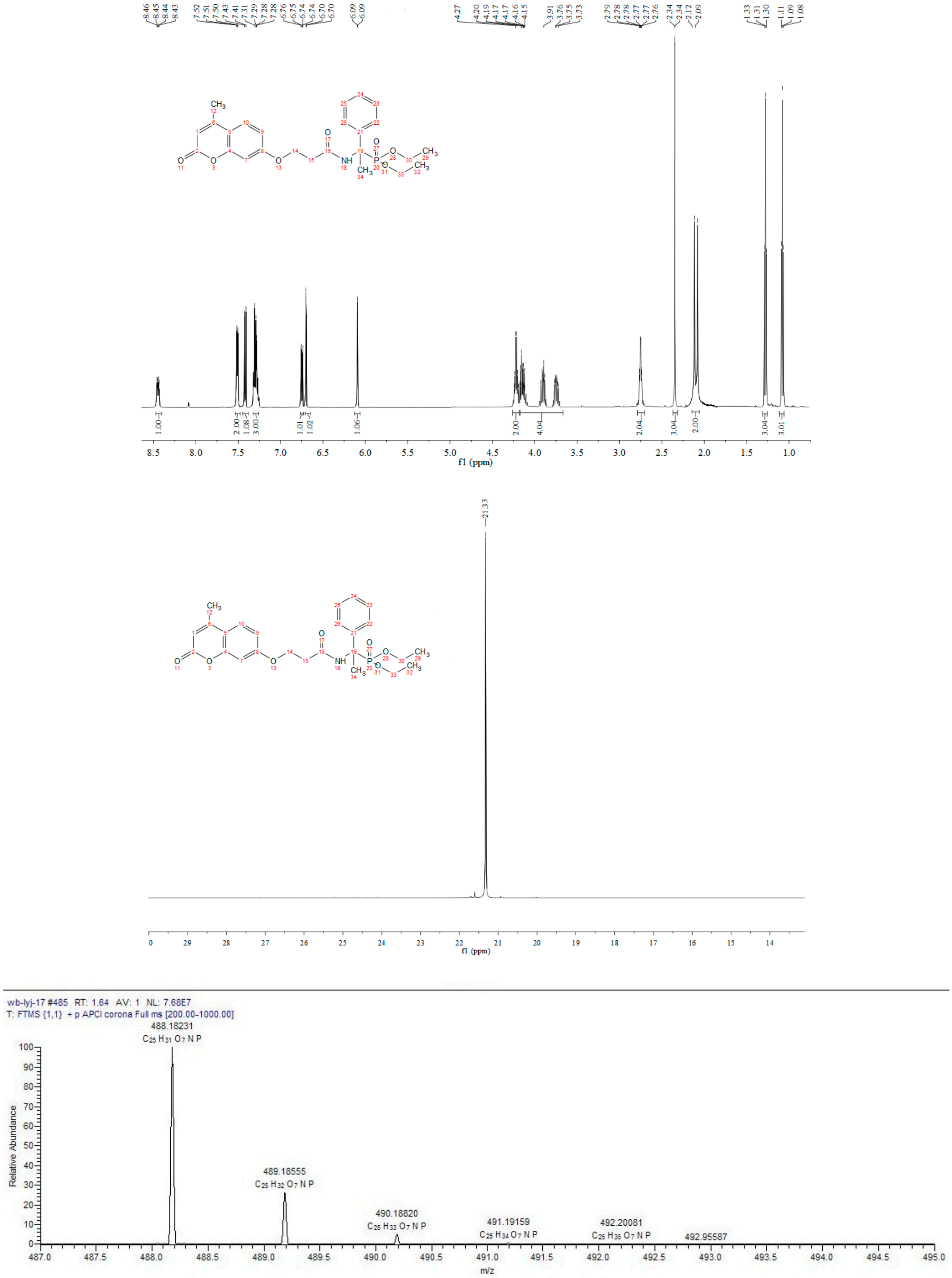


**Figure S45.** Spectrum of compound **8j**.
